# Biomedical applications of stimuli‐responsive nanomaterials

**DOI:** 10.1002/mco2.643

**Published:** 2024-07-20

**Authors:** Xiaojie Chen, Di Wu, Zhong Chen

**Affiliations:** ^1^ Key Laboratory of Neuropharmacology and Translational Medicine of Zhejiang Province School of Pharmaceutical Sciences Department of Neurology The First Affiliated Hospital of Zhejiang Chinese Medical University (Zhejiang Provincial Hospital of Chinese Medicine) Hangzhou China

**Keywords:** biomedical applications, drug delivery, imaging, pathological environment, stimuli‐responsive nanomaterials, theranostics

## Abstract

Nanomaterials have aroused great interests in drug delivery due to their nanoscale structure, facile modifiability, and multifunctional physicochemical properties. Currently, stimuli‐responsive nanomaterials that can respond to endogenous or exogenous stimulus display strong potentials in biomedical applications. In comparison with conventional nanomaterials, stimuli‐responsive nanomaterials can improve therapeutic efficiency and reduce the toxicity of drugs toward normal tissues through specific targeting and on‐demand drug release at pathological sites. In this review, we summarize the responsive mechanism of a variety of stimulus, including pH, redox, and enzymes within pathological microenvironment, as well as exogenous stimulus such as thermal effect, magnetic field, light, and ultrasound. After that, biomedical applications (e.g., drug delivery, imaging, and theranostics) of stimuli‐responsive nanomaterials in a diverse array of common diseases, including cardiovascular diseases, cancer, neurological disorders, inflammation, and bacterial infection, are presented and discussed. Finally, the remaining challenges and outlooks of future research directions for the biomedical applications of stimuli‐responsive nanomaterials are also discussed. We hope that this review can provide valuable guidance for developing stimuli‐responsive nanomaterials and accelerate their biomedical applications in diseases diagnosis and treatment.

## Graphical abstract

In this review, we summarize the responsive mechanisms of a variety of endogenous and exogenous stimulus and discuss their biomedical applications, as well as the remaining challenges and outlooks of future research directions for the biomedical applications of stimuli‐responsive nanomaterials.

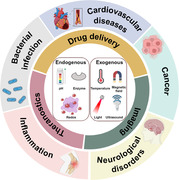

## 1 INTRODUCTION

As one of the emerging fields in 21st century, nanotechnology that integrates the basic attributes of biological, physical, and chemical sciences has attracted much attention in the field of medicine.^1^, ^2^ Owing to its unique nanoscale structure, ease to modify, and multifunctional physicochemical properties, nanotechnology has been demonstrated enhanced drug loading, stability, tissue‐targeting, and blood circulation, as well as minimized side effects compared with small molecules.^3^ Particularly, nanomaterials, such as nanoparticles, polymers, liposomes, and micelles, are regarded as promising avenues for biomedical applications including drug delivery, bio‐imaging, diagnosis, and therapy.^4^, ^5^, ^6^ However, the applications of nanomaterials are still hampered by certain challenges, including uncontrolled drug release, nonspecific biodistribution, and off‐target effects, which largely limit the therapeutic effectiveness.^7^

Recently, there has been a growing focus on stimuli‐responsive nanomaterials (also known as “smart” nanomaterials) equipped with stimuli‐triggered modules for drug delivery.^8^, ^9^ In contrast to traditional nanomaterials that are developed as drug carriers to release payloads upon reaching the targeting site, stimuli‐responsive nanomaterials are defined as a type of nanomaterials who are able to liberate drug payloads in response to either endogenous or exogenous stimulus, exhibiting “on‐off” functionalities.^8^, ^10^, ^11^ These nanomaterials can be induced by pH, enzyme, and redox conditions within microenvironment, as well as exogenous stimulus such as thermal effect, magnetic force, light, and ultrasound stimulation.^4^, ^12^, ^13^ One of the ultimate objectives of stimuli‐responsive nanomaterials is to precisely control drugs release and minimizing the toxicity of drugs toward normal tissues.^14^, ^15^

In conjunction with the in‐depth investigation of stimuli‐responsive nanomaterials, numerous reviews also have summarized the relative progress of stimuli‐responsive hydrogels, polymers, peptide assemblies, nanozymes, and liposomes for biomedical applications.^16^, ^17^, ^18^, ^19^, ^20^, ^21^ For instance, Du et al.^22^ discussed the design and development of stimuli‐responsive nanoparticles for targeting tumor therapy. Cai et al.^23^ and Wang et al.^24^ reviewed the metal‐organic frameworks‐based stimuli‐responsive nanomaterials for drug delivery. In addition, stimuli‐responsive nanomaterials in cancer treatment were summarized by a significant number of reviews, which mainly owing to the complexity of tumor microenvironment (TME). However, a comprehensive review applying on stimuli‐responsive nanomaterials for biomedical applications is still lacking.

**FIGURE 1:**
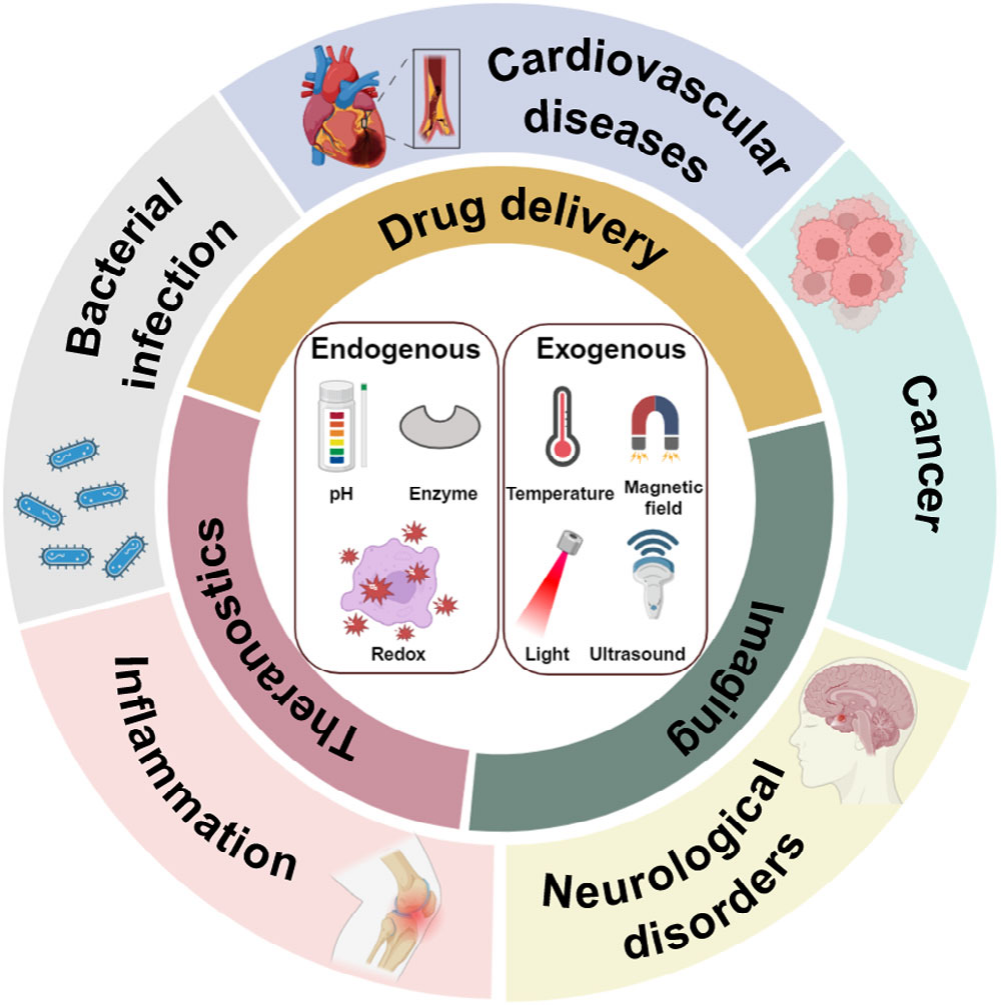
Biomedical applications of stimuli‐responsive nanomaterials (created with BioRender.com).

Hence, to better utilize the specific advantages of nanomaterials in a number of biomedical applications such as cardiovascular diseases (CVDs), cancer, neurological disorders, inflammation, and bacterial infection, this review will focus on the design and the triggered mechanism of stimuli‐responsive nanomaterials based on endogenous or exogenous stimulus. Additionally, we present recent advancements in biomedical applications of stimuli‐responsive nanomaterials are presented, and the prospects for the development of these nanomaterials is also discussed. We anticipate that this review will provide a guided and updated reference for researchers working within materials science and biomedicine (Figure 1).

## 2 STIMULI‐RESPONSIVE NANOMATERIALS

In recent years, stimuli‐responsive nanomaterials have garnered wide attention in the community of biomedicine and nanotechnology due to their tailored release capabilities with excellent spatial, temporal, and dosage control. These nanomaterials are engineered based on their sensitivity to specific endogenous factors (i.e. pH, redox, enzyme, and etc.) or exogenous stimulus (i.e. thermal changes, magnetic field, light, ultrasound, and etc.). In this section, we will summarize the materials design principles and responsive mechanisms of stimuli‐responsive nanomaterials (Table 1).

**TABLE 1:** Summary of stimuli‐responsive nanomaterials and their advantages and disadvantages.

Stimuli	Nanomaterials	Mechanisms	Advantages	Disadvantages	Animal models	References
pH	Copolymer micelles conjugated with DOX and NIR probe	pH‐sensitive chemical bond cleavage (hydrazone bonds)	The pH‐responsive copolymer micelles have excellent biocompatibility and low toxicity.	The limited pH‐responsive ability of hydrazone bond may hamper the drug release and anticancer effect of nanomaterials.	No data	25
pH	APCI	Protonation	APCI displayed good stability in physiological environment and high drug loading efficiency of 62%. The pH‐responsive properties of APCI might be due to the protonation of sulfonic groups of IR783 in acid condition.	Owing to their intricate nature and high specificity, the clinical translation of self‐assembled nanomaterials is challenging.^14^	LN229 glioma cells‐bearing mice (orthotopic GBM)	26
pH	FAND	Protonation	The metal coordination bonds in FAND were cleaved by the protonation of the carboxyl groups. The pH‐responsive release behavior can minimize the premature leakage‐induced toxicity to enhance antitumor effect.	The tumor‐targeting ability and in vivo toxicity may hamper the clinical applications.	A549‐bearing mice (xenograft lung cancer)	27
pH	Ang–RBCm–CA/siRNA	Charge conversion	The charge conversion of CA was triggered by the acid environment of tumor cell endo/lysosome, and the RBCm collapsed, further enhancing siRNA release.	The clinical application of nanomaterials remains challenging owing to their preparation and cost.	U87MG tumor‐bearing mice (orthotopic GBM)	28
pH	Chitosan	Protonation	Chitosan has excellent biocompatibility, biodegradability, and nontoxicity properties and exhibits a pH‐responsive behavior due to the amino groups on chain.	Chitosan is insoluble at high pH, whereas dissolves easily at lower pH values.	No data	29, 30, 31
Redox	PSNPs	Disulfide bond (GSH)	GSH triggers the dissociation of disulfide bonds PTX prodrug to release PTX, which could avoid drug leakage and enhance the antitumor efficiency.	The clinical translation of drug self‐delivery systems is limited in their stability and targeting ability.	U87MG tumor‐bearing mice (orthotopic glioma)	32
Redox	LSN	Disulfide bond (GSH)	The excess GSH in tumor site could efficiently cleave the disulfide bond in dimer, and release LND and NLG919 for destroying mitochondria and alleviating the immunosuppressive, respectively.	The stability and drug release efficiency of nanomaterials may influence the therapeutics.	4T1 tumor‐bearing mice (xenograft breast cancer)	33
Redox	DOX@MSN‐S‐S‐RGD	Disulfide bond (GSH)	GSH triggered the cleavage of disulfide bonds in DOX@MSN‐S‐S‐RGD by intracellular GSH to release DOX, thereby enhancing the antitumor efficacy.	The in vivo toxicity and nondegradability of MSN impede its biomedical application.	No data	34
Redox	Ap‐CSTKSA/R complexes	ROS‐reactive linker (TK)	ROS triggered the release of siRNA, as well as promoted the release siRNA escaping from endosomes.	The intrinsic ROS concentration in the microenvironment is too low to cleave TK.	U87 tumor‐bearing mice (orthotopic GBM)	35
Redox	MPEG–(TK‐CPT)–PPa	ROS‐cleavable dual prodrug (TK)	Under laser irradiation, PPa can not only generated ROS to cleave TK ligand to release CPT, but also endowed nanomaterials with precisely fluorescence imaging.	The clinical application of ROS generation mediated by laser irradiation will be limited in the tissue penetration depth of laser.	HCT116 tumor‐bearing mice (xenograft colon cancer)	36
Redox	MnO_2_	GSH/ROS	MnO_2_ can be served as a gatekeeper for controlling drug release, reduce the toxicity of Mn^2+^, and act as the contrast agent for MRI. Furthermore, this process will consume GSH, and convert H_2_O_2_ into O_2_ and ·OH to enhance antitumor effect.	The biosafety and metabolism of inorganic nanomaterials should be concerned.	U87MG tumor‐bearing mice (xenograft glioma)/CT26 tumor‐bearing mice (xenograft colon cancer)	37, 38, 39, 40
Enzyme	GNP–DOX/ICG	MMP‐2 responsive nanosystem	Owing to NIR‐mediated swelling and MMP‐2‐responsive degradation properties of gelatin, GNP–DOX/ICG can achieve tumor accumulation and deeper penetration of drugs, further improving antitumor efficiency.	The overexpressed MMP‐2 is not only in the cells, but also in extracellular. Improving the accurately intracellular degradation of nanomaterials is essential for enhancing effects.	4T1 tumor‐bearing mice (xenograft breast cancer)	41
Enzyme	T‐mPDA–Pep–Mino	MMP‐2 responsive peptide	The MMP‐2 responsive peptide triggers the on‐demand release of Mino in the ischemic region, thereby improving circulation time and reduce the toxicity of Mino.	The cost of enzyme‐responsive peptides is exorbitant.	Ischemic stroke (MCAO)	42
Thermal	PES–Au@PDA NPs	Photothermal effect	Owing to the good photothermal properties of PDA, PES–Au@PDA NPs enables hyperthermia‐responsive release of PES, leading to remarkably promote the synergistic radiophotothermal therapy efficiencies.	The clinical applications of NIR are hampered by the limited tissue penetration depth.	SW1783 tumor‐bearing mice (xenograft GBM)	43
Thermal	DOX@P1NS/TNC–FeLPs	Thermal‐responsive lipids	Applying an AMF to transform electromagnetic energy to heat, it can effectively control the drug release under mild hyperthermia.	The clinical application is challenging owning to the intricate nature of materials preparation.	No data	44
Magnetic	GRGDS–Cur‐m‐PNPs	RF–HT	Magnetic nanoparticle‐mediated hyperthermia can enhance the sensitivity of cancer cells and release drugs in a controlled manner.	The utilization of RF–HT will be hindered by the low specificity and potential side effects to health tissues.	No data	45
Magnetic	DOX–CS–MNPs	Magnetic Fe_3_O_4_	The magnetic‐responsive aggregation and pH‐responsive release behavior of DOX–CS–MNPs can efficiently inhibit the proliferation of tumors.	In vivo biosafety evaluations and metabolism manner of magnetic nanomaterials are neglected.	No data	46
Light	cRGD–HN–DOX	NIR‐responsive	Under mild NIR irradiation, the drug release from cRGD–HN–DOX was markedly accelerated, thereby significantly enhancing antitumor effects.	The in vivo tissue penetration of depth is limited for clinical applications.	U87MG tumor‐bearing mice (xenograft GBM)	47
Ultrasound	TSL–DOX	MRgFUS‐hyperthermia	Utilizing the MRgFUS‐hyperthermia as the stimulus, it could efficiently attain local hyperthermia within a desired temperature range, as well as promote the drug release.	The clinical application might be hindered by the targeting ability of nanomaterials and the accumulation of drugs at specific sites.	GL261 tumor‐bearing mice (orthotopic GBM)	48
Multiple stimuli	Curcumin and quercetin loaded FCS/HA NPs	pH/ROS	Chitosan functionalized with ROS‐responsive weak acid displayed pH/ROS dual stimuli‐responsive property to release curcumin and quercetin, result in an accelerated drugs release in a desired manner.	The dual‐responsive nanomaterials require more complex preparation technologies.	No data	49
Multiple stimuli	DOX–ANG–CMCSN	pH/GSH	The DOX–ANG–CMCSN exhibited good pH and reduction sensitivity and accelerate drug release with the combined stimulus.	The clinical application remains challenging due to the complex preparation.	C6 tumor‐bearing mice (xenograft GBM)	50
Multiple stimuli	CTHG–Lf NPs	pH/enzyme/light	Multistimuli responsive nanomaterials can not only promote drug delivery and responsive release at tumor site, but also effectively suppress the tumor growth.	The clinical application will be limited by the intricate nature of preparation. Moreover, multiple stimulus should be combined to maximize their own benefits.	C6 tumor‐bearing mice (orthotopic GBM)	51

### 2.1 pH‐responsive nanomaterials

Among the nanomaterials triggered by endogenous stimuli, pH‐responsive nanomaterials are extensively exploited owing to the pH variations in specific organs (stomach with a pH range of 1−3) or pathological microenvironment, such as tumor tissue, bacterial infection, and inflammation.^52^ In general, pH in normal tissues is around 7.4, whereas that in TME is at mild acidity (pH 6.5–7.0) attributed to high metabolic activity and inadequate perfusion.^53^ A relatively lower pH value could be observed after internalized into endo/lysosomes (pH 5.0–6.5).^10^, ^28^ Therefore, the acidic pH could be harnessed for nanomaterials as a potential trigger for specific drug release in pathological tissues through mechanisms including (1) pH‐sensitive bonds or linkers, (2) pH‐triggered charge conversion, and (3) pH‐responsive carriers.

#### 2.1.1 pH‐sensitive bonds or linkers

A number of pH‐responsive nanomaterials have been developed in response to the acidic microenvironment based on their physicochemical properties. The potential mechanisms of these nanomaterials could be mainly classified as the cleavage by protonation of chemical groups and pH‐sensitive bonds/linkers, including hydrazone bonds, imine bonds, ester bonds, amide bonds, metal ion coordination bonds, and noncovalent interactions (e.g., hydrogen bonds, π–π stacking, and electrostatic forces).^54^, ^55^, ^56^, ^57^, ^58^, ^59^, ^60^, ^61^, ^62^, ^63^, ^64^, ^65^ These pH‐sensitive bonds are comparatively stable at physiological condition, but could break down under acid condition, further triggering the drug release from nanomaterials.

Hydrazone bonds could serve as an ideal linkage by formation of hydrazine and aldehyde/carbonyl groups. Compared with other pH‐sensitive bonds, hydrazone bonds are more stable under physiological condition, but will rapidly hydrolyze in acid environment of endo/lysosomes.^66^ Doxorubicin (DOX) is a broad‐spectrum anticancer drug containing carbonyl groups and can be chemically conjugated with hydrazide groups on polymer or pectin through hydrazone bonds formation.^67^, ^68^ After self‐assembling into micelles, approximately 65% of DOX was released from copolymer within 72 h at pH 5.4, whereas only 31% at neutral condition.^25^ Lu et al.^26^ purposefully developed a multicomponent self‐assembly nanocomplex (Ang–PEG‐g‐PLL@CPT–RT@IR783; APCI) through electrostatic, π–π stacking and hydrophobic interactions, which were self‐assembled toluenesulfonyl protected arginine‐conjugated camptothecin (CPT–RT) with canine dyes (IR783). Due to the protonation of sulfonic groups of IR783 under acidic conditions, a large amount of CPT–RT could be released from APCI at pH 5.0 after 24 h compared with that in physiological environment and exhibited a pH‐responsive drug release property.

Recently, metal ion coordination bonds have been investigated for the design of pH‐responsive nanomaterials. Metal ions, such as manganese, iron, or calcium, can self‐assemble with negative ligands to form coordination bonds, which could be attributed to the competitive binding between metal ions and protons.^69^, ^70^, ^71^ Driven by metal ion coordination as well as noncovalent forces, a full‐active pharmaceutical ingredient nanodrug (FAND) was designed. FAND kept excellent stability under physiological conditions, with only 12.8% of drugs was released over 12 h. But the cumulative release was dramatically enhanced to 75.5% at pH 5.0, owning to the protonation of carboxyl group and cleavage of metal ion coordination bonds.^27^

#### 2.1.2 pH‐triggered charge conversion

As the cell membrane is negatively charged, nanomaterials with positive charge exhibit enhanced cellular uptake efficiency via electrostatic interactions compared with nanomaterials with negative charge. However, an excessively positive charge would increase cytotoxicity and decrease stability, as well as lower systemic circulation time.^72^ Therefore, it is valuable to design pH‐responsive nanomaterials with variable surface charges for sensing pH variations between normal and diseased tissues.

**FIGURE 2:**
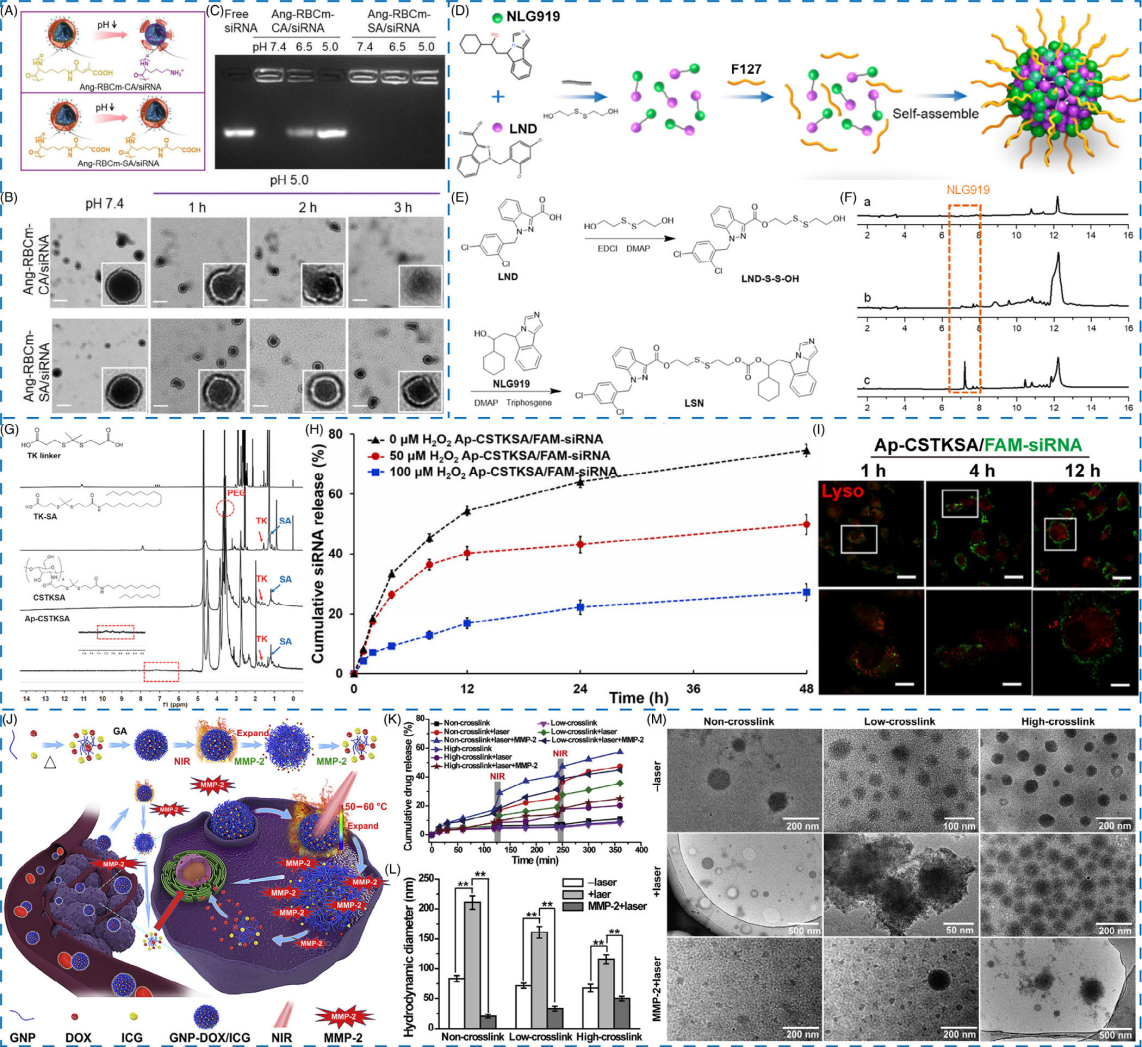
Endogenous stimuli‐responsive nanomaterials. (A) Schematic illustration of the pH‐responsive mechanism of Ang–RBCm–SA/siRNA. (B) TEM images of membrane change at pH 7.4 or 5.0. (C) Gel retardation assay at various pH values. (A–C) Reproduced with permission from Liu et al.^28^ Copyright 2020, American Chemical Society. (D) Schematic illustration of the GSH‐responsive dimeric prodrug. (E) Synthetic route of LSN. (F) The GSH‐responsive property of LSN. (D–F) Reproduced with permission from Liu et al.^33^ Copyright 2021, American Chemical Society. (G) ^1^H NMR spectra of ROS‐responsive linkages. (H) In vitro siRNA release of nanoparticles with various concentration of H_2_O_2_. (I) Colocalization of nanoparticles with endo‐lysosomes in U87 cells. (G–I) Reproduced with permission from Wen et al.^35^ Copyright 2022, Spring Nature. (J) Schematic illustration of the preparation of GNP–DOX/ICG and its MMP‐2/NIR‐responsive properties. (K) TEM images and (L) hydrodynamic diameter, and (M) drug release manner of GNP–DOX/ICG in the presence or absence of MMP‐2 or laser. (J–M) Reproduced with permission from Chen et al.^41^ Copyright 2021, Elsevier BV.

Charge‐conversion strategy has been constructed for drug delivery, inspired by the pH‐triggered charge reversal from negative or neutral charge under physiological environment to positive charge at acidic condition.^28^, ^73^, ^74^ By incorporating acylsulfonamide‐based pH‐responsive zwitterionic ligands on the surface of nanomaterials, the formed pH‐responsive nanomaterials can reverse charge in response to the decreased pH at diseased site, but the structure will not be altered.^74^ Liu et al.^28^ constructed a charge conversional biomimetic nanoplatform (Ang–RBCm–CA/siRNA) by leveraging Angiopep‐2 peptide, red blood cells membrane, and citraconic anhydride grafted poly‐L‐lysine (PLL‐CA) as charge conversional component for siRNA delivery. Owing to the charge conversion ability of CA, the membrane of biomimetic nanoplatform Ang–RBCm–CA/siRNA was gradually destroyed with the pH decreased and time prolonged, but the nonsensitive nanoplatform remained a spherical structure under both conditions (Figure 2A,B). Agarose gel electrophoresis assay proved that siRNA was released from Ang–RBCm–CA/siRNA at pH 5.0, further demonstrating the pH responsive and charge‐conversional behavior of CA (Figure 2C).

#### 2.1.3 pH‐responsive carriers

Polymers containing H^+^ labile linkages will occur protonation under lower pH conditions, leading to a change of physical structures and subsequent release of loaded drugs. Polymers capable of reversible ionization with amino and carboxylic groups are commonly used as pH‐responsive carriers for drug delivery, such as alginate, hyaluronic acid (HA), carboxymethylcellulose, and chitosan.^75^ For example, chitosan, a pH‐responsive polymer, is proved to have excellent biodegradable, biocompatible, and nontoxic properties.^76^ Under physiological environment, chitosan is insoluble, whereas the amino groups within chitosan become protonated and soluble under acidic pH condition, further promoting drug release.^29^, ^30^, ^31^

In addition to pathological microenvironment, physiological environment might be acid as well, such as the stomach. Oral administration is a commonly used medication strategy in clinical practice due to its low cost, simplicity, and convenience. For oral administration systems, they are supposed to protect drugs from harsh conditions of gastrointestinal tract, enhance absorption into circulation system, target specific sites, and achieve controlled release.^77^ One design approach is to prepare pH‐responsive swelling nanomaterials based on the carboxyl protonation. Surface‐functionalization with acid‐stable targeting ligands (e.g. polyanions, polycations and inorganic materials) can also improve the stability of nanomaterials.^78^

Overall, through surface modification of nanomaterials, pH‐sensitive bonds or linkers can be conjugated with polymers to endow nanomaterials with pH‐responsive properties. Upon exposure to pathological environment, these nanomaterials will occur cleavage, protonation, or charge conversion in response to altered pH conditions. Besides, a variety of pH‐responsive carriers and derivatives have been developed to facilitate insoluble‐to‐soluble transformation at acid conditions, leading to controlled release of loading drugs. However, pH‐responsive nanomaterials have not been approved for clinical applications. Several challenges of these nanomaterials should be overcome, such as the pH heterogeneity in the tumor site, complex preparation technology, and systemic toxicity of nanomaterials.^55^ Therefore, to facilitate further translational study on pH‐responsive nanomaterials, the nontoxicity, simple preparation, and specificity of nanomaterials should be taken into consideration.

### 2.2 Redox‐responsive nanomaterials

Redox potential levels are closely associated with the progression of diseases, making redox‐responsive nanomaterials highly attractive for drug delivery and controlled release.^79^, ^80^ Glutathione (GSH) and reactive oxygen species (ROS) are overabundant antioxidant cofactors, which synergistically maintain intracellular redox homeostasis.^81^, ^82^ In this section, we will discuss the mechanisms of redox‐responsive nanomaterials focusing on GSH‐responsive and ROS‐responsive releases.

#### 2.2.1 GSH‐responsive nanomaterials

As a ubiquitous reductive agent, GSH is a tripeptide capable of antioxidant properties and other vital functions, including redox homeostasis, cell proliferation detoxification, and so on.^83^ It has been reported that GSH level is higher in diseased tissues than that in healthy ones, especially elevated in tumors, which can achieve a fourfold higher concentration (2–10 mM) than in normal tissues.^84^ Disulfide bonds (S─S), prone to be stable under physiological environment, but swiftly cleaved in the presence of GSH, are widely applied to prepare GSH‐responsive nanomaterials.^85^, ^86^, ^87^, ^88^, ^89^ The cleavage mechanism of GSH‐responsive nanomaterials under high GSH concentration relies on the thiol‐disulfide exchange reaction between disulfide bonds and free thiols of GSH.^90^

Disulfide bonds can work as GSH‐responsive ligands linking drug molecules to form nanoprodrugs, such as paclitaxel (PTX), CPT, and DOX.^32^, ^91^, ^92^ Jiang et al.^32^ proposed PTX‐SS‐C_18_‐conjugated self‐assembled nanoparticles (PSNPs) for glioblastoma (GBM) treatment. PTX‐SS‐C_18_ was synthesized by mixing dithiodiglycolic acid, anhydrous acetic anhydride, N,N‐diisopropylethylamine, and PTX. ^1^H NMR and MS spectra showed that octadecanol was conjugated with PTX via S‐S. After incubation with 10 mM GSH, the spherical morphology and size distribution demonstrated the disassembly of the nanoparticles. Likewise, PTX could be released rapidly from nanoparticles in response to elevated GSH in the tumor site.

Several prodrugs were synthesized by introducing a disulfide bond between two different drug molecules to regulate the corelease of both drugs and realize an enhanced antitumor effect.^33^, ^93^ For example, the dimer of lonidamine and NLG919 was connected via a disulfide bond, which could be cleaved by GSH to realize drug release (Figure 2D,E). To enhance the transport of the dimer, Pluronic F127 was chosen to promote the formation of nanoprodrugs (LSN). When encountered with GSH, the disulfide bond in the dimer was broken, leading to the release of two drug molecules for their respective functions in cancer treatment (Figure 2F).^33^

Additionally, the doping of disulfide bonds will confer nanomaterials with GSH‐responsive biodegradability, and lower their potential toxicity.^94^ The surface disulfide linker on mesoporous silica nanoparticles (MSN‐S‐S‐NH_2_) was introduced by mercaptopropyl‐derivatized MSN reacting with S‐(2‐aminoethylthio)‐2‐thiopyridine hydrochloride. After that, RGD peptide was capped onto MSN using click chemistry for tumor‐targeting and controlled drug release. Under reductive conditions, over 33 and 78% of DOX were released within 90 min at the GSH concentration of 2 and 10 mM, respectively, but less than 18% was released over 24 h in the absence of GSH.^34^

#### 2.2.2 ROS‐responsive nanomaterials

Biologically, as secondary messengers in modulating cellular functions, ROS is a class of oxygen‐containing molecules, including hydrogen peroxide (H_2_O_2_), hydroxyl radicals (∙OH), singlet oxygen (^1^O_2_), and superoxide (O_2_^−^). Distinct from the physiological environment, the concentration of ROS is overproduced and up to around 50−100 μM at pathological conditions, such as in tumor and inflammation. The disparity in ROS concentration between healthy and diseased tissues has fueled the development of nanomaterials with ROS‐responsive ability for targeting diseased regions and minimizing the toxicity to normal tissues. Hence, numerous ROS‐responsive nanomaterials have been developed by using ROS‐responsive linkages, such as thioethers or sulfides, thioketal (TK), phenylboronic ester group for targeted and controllable drug release.^35^, ^95^, ^96^, ^97^, ^98^

TK is a biodegradable and nontoxic thioether group, which can be incorporated into the nanostructure or serve as a ROS‐reactive linker for ROS‐responsive nanomaterials.^7^, ^99^, ^100^, ^101^ The stability of the TK group in physiological condition allows for its easy cleavage under oxidative environment. By taking the ROS‐responsive property of TK, Wen et al.^35^ constructed an angiopep‐2 peptide modified and ROS‐cleavable nanocarrier for siRNA delivery. The TK linker was prepared by mixing 3‐mercaptopropionic acid with anhydrous acetone, following with stirring, crystallizing, and drying. The results showed that high concentration of H_2_O_2_ could significantly enhance the cumulative release of siRNA from the complexes and promote siRNA escaping from endosomes through the ROS‐responsive drug release effect, confirming the excellent ROS‐responsive effect of TK (Figure 2G–I).

However, due to the low intrinsic concentration of ROS, Chu et al.^36^ developed an ROS‐cleavable dual prodrug self‐assembly nanoparticle via conjugating CPT with poly(ethylene glycol) methyl ether (MPEG) by TK linkage. Photosensitizer pyropheophorbide‐a (PPa) was connected to MPEG via lipid linkage for ROS generation through photodynamic therapy (PDT). With the assistance of laser illumination, the released CPT from MPEG–(TK‐CPT)–PPa exhibited enhanced performance, and about 40.4% CPT was released from the nanoparticles within 48 h. Besides, the addition of extrinsic ROS source through Fenton reaction via Fe^2+^ and H_2_O_2_ further enhanced the release of CPT. These results showed that ROS is an effective endogenous triggered factor for controlling drug release and can bridge the link between nanomaterials and drugs for disease treatment.

#### 2.2.3 GSH/ROS dual responsive nanomaterials

Besides, GSH/ROS dual responsive nanocarriers such as manganese dioxide (MnO_2_), PTX‐TKN, and PMPC‐P (Se‐co‐RB)‐P can also serve as smart materials to control drug release and protect the drugs from premature leaking.^102^, ^103^ As a typical TME dual‐responsive nanocarrier, MnO_2_ has aroused widespread attention in cancer treatment.^37^, ^38^ Through biocompatible functionalization, the toxicity induced by manganese ion (Mn^2+^) could be reduced in MnO_2_ nanomaterials.^104^ Notably, MnO_2_ can not only undergo a redox reaction in the presence of high GSH concentration of TME to yield Mn^2+^ and simultaneously consume GSH, but also convert H_2_O_2_ into oxygen (O_2_) and ·OH through the Mn^2+^‐mediated Fenton‐like reaction.^39^, ^40^ Therefore, MnO_2_‐based nanomaterials with good biocompatibility and dual GSH/ROS‐responsive ability make them to promisingly applicable in cancer treatment.^105^, ^106^, ^107^

### 2.3 Enzyme‐responsive nanomaterials

Owing to their high efficiency, exceptional sensitivity, and outstanding catalytic properties, enzymes play an important role in the majority of biochemical and biological processes in bodies.^108^ However, the expression and activity of these enzymes could be up‐ or downregulated in various pathological conditions, such as cancer and inflammation.^109^ Hence, the design of enzyme‐responsive nanomaterials is a potential strategy for controlling drug release at the desired biological target.^110^ Up to now, several enzymes, such as proteases, phospholipases, and oxidoreductases, have been widely exploited as stimuli for enzyme‐responsive nanomaterials construction.

Matrix metalloproteinases (MMPs) are a class of enzymes for extracellular matrix components degradation and cell signals regulation.^111^ MMP‐2 and MMP‐9 have been mostly employed for responsive cleavage of MMP substrates (e.g., collagen, gelatin, fibrinogen) and well‐designed peptides for on‐demand drug release.^112^, ^113^, ^114^, ^115^, ^116^ As a naturally derived polymer from collagen hydrolysis, gelatin possesses inherent advantages such as biodegradability, biocompatibility, and nontoxicity.^117^ The excellent MMP‐2‐responsive ability of gelatin has aroused extensive attentions in cancer treatment. In previous study, Chen et al.^41^ have proposed an MMP‐2‐responsive nanosystem (GNP–DOX/ICG) for codelivery of DOX and indocyanine green (ICG) in breast cancer treatment (Figure 2J). Glutaraldehyde was added to improve mechanical ability and stability of gelatin. GNP–DOX/ICG exhibited obvious degradation in the presence of MMP‐2, which was observed through changes of morphology and hydrodynamic diameter (Figure 2L,M). In addition, with the assistance of laser irradiation, MMP‐2‐stimulation resulted in 44.8% of DOX released from low‐crosslinked GNP–DOX/ICG after 6 h, which was higher than without MMP‐2 addition (35.7%) (Figure 2K).

MMP‐2 is also overexpressed in the inflammation environment. Wu et al.^42^ have designed an MMP‐2‐responsive peptide (Ac‐CSSSGPLGIAGQSSS) to connect drugs and nanocarriers for ischemic stroke (IS) treatment. MMP‐2‐responsive peptide was conjugated with minocycline through esterification reaction as a cleavable cross‐linker and modified on the surface of mesoporous polydopamine (PDA) under alkaline solution. This nanomaterial was broken up by MMP‐2, releasing minocycline to inhibit proinflammatory polarization of microglia.

These works highlight the potential of enzyme‐responsive drug release, but the concentration and activity of enzymes in pathological environment are still needed to be further investigated. Therefore, it is worthwhile to design more sensitive enzyme‐responsive nanomaterials tailored to specific pathological environment.

### 2.4 Thermal‐responsive nanomaterials

Although endogenous stimulus is a promising strategy for on‐demand drug release, it is difficult to control spatiotemporally in high resolution due to the rapid variation of pathological environment in patients. In addition to endogenous stimulus, exogenous triggers can also be employed as a switch for on‐demand drug release, including thermal changes, magnetic field, light, and ultrasound.

Thermal‐responsive nanomaterials are one of the most widely explored exogenous stimuli‐triggered systems in biomedical applications, which often integrate with light or ultrasound stimuli.^8^ These nanomaterials are generally dependent on the uses of thermo‐responsive materials (such as liposomes, polymers, or nanoparticles) or photothermal conversion agents, thus modulating the drug release in response to the change of temperature.^118^, ^119^, ^120^, ^121^, ^122^ Ideally, thermal‐responsive materials should remain stable at body temperature (∼37°C), while undergoing a triggered drug release after local hyperthermia within the desired site (∼41–43°C).^9^ Typically, the temperature should be kept at less than 43°C to prevent damages to normal tissues.

**FIGURE 3:**
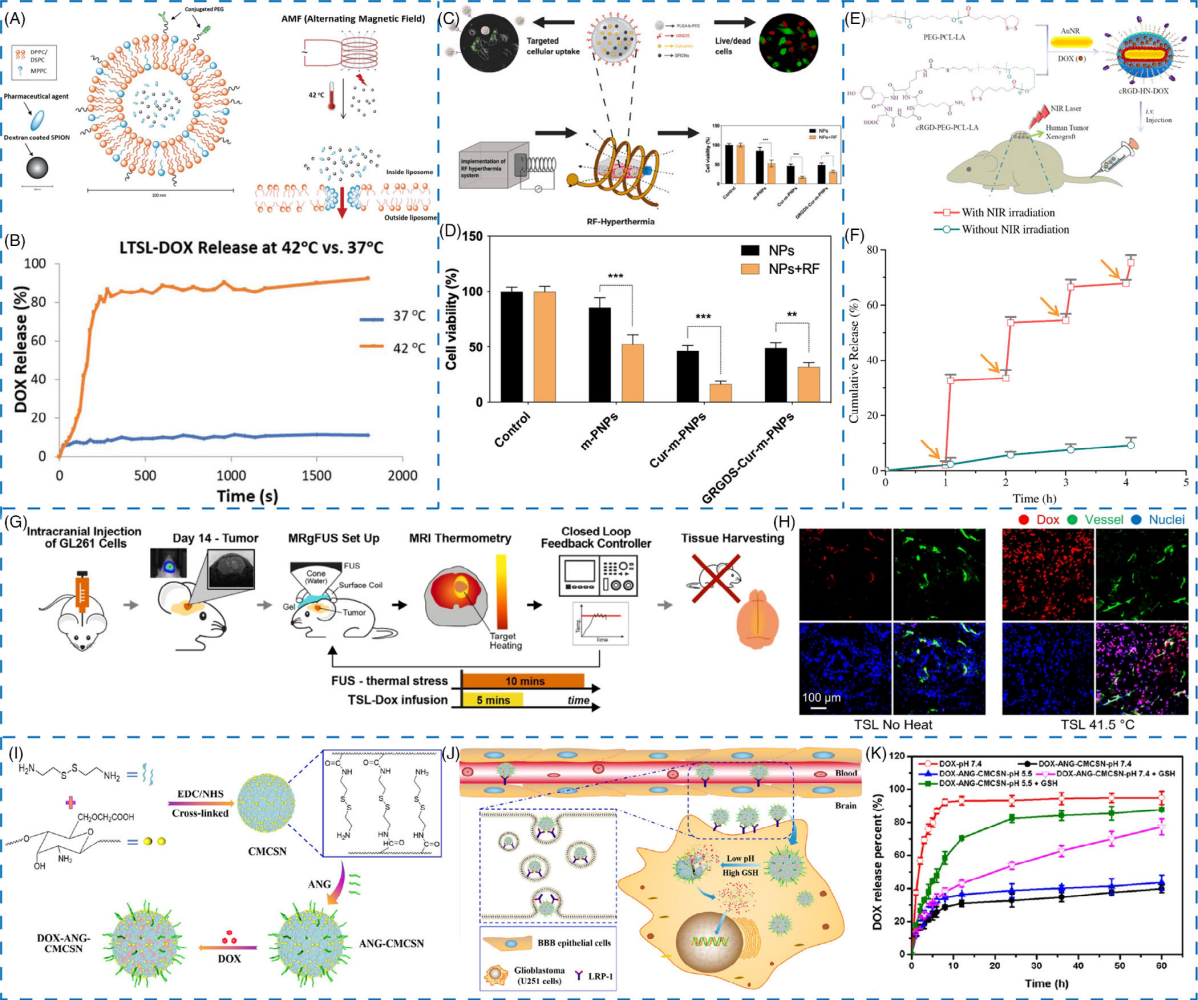
Exogenous stimuli‐responsive nanomaterials. (A) Schematic illustration of AMF induced drug release. (B) Percentage of DOX release from liposome at 42 vs. 37°C. (A and B) Reproduced with permission from Shi et al.^44^ Copyright 2019, The Royal Society of Chemistry. (C) Schematic illustration of the RF–HT system of GRGDS–Cur‐m‐PNPs for GBM treatment. (D) Cytotoxicity of NPs and NPs+RF treatment. (C and D) Reproduced with permission from Senturk et al.^45^ Copyright 2021, Elsevier BV. (E) Schematic illustration of the preparation, tumor targeting and drug release of cRGD–HN–DOX. (F) In vitro NIR‐triggered DOX release. (E and F) Reproduced with permission from Zhong et al.^47^ Copyright 2014, Elsevier BV. (G) Schematic illustration of the experimental protocol. (H) Fluorescence images of DOX in the tumor after various treatments. (G and H) Reproduced with permission from Kim et al.^48^ Copyright 2021, Ivyspring International Publisher. Schematic illustration of the (I) preparation of DOX–ANG–CMCSN and (J) pH/GSH‐responsive drug release process. (K) Responsive release behavior of DOX–ANG–CMCSN. (I–K) Reproduced with permission from Song et al.^50^ Copyright 2021, American Chemical Society.

For instance, Zhu et al.^43^ designed a bio‐responsive nanoplatform to deliver a heat shock protein A5 inhibitor (pifithrin‐μ; PES) and a radiosensitizer (gold nanosphere; AuNS) by using PDA as the photothermal conversion agent for GBM therapy. The PES–Au@PDA nanoparticles exhibited excellent photothermal effect and stability, along with thermal‐responsive drug release at elevated temperatures owing to the destruction of π–π stacking interaction. Moreover, two thermal‐responsive lipids of dipalmitoylphosphatidylcholine and 1,2‐distearoyl‐*sn‐glycero*‐3‐phosphocholine were used to construct the original thermal‐responsive liposomes giving a gel‐to‐liquid transition at the temperature of 41−43°C.^44^ In another report, superparamagnetic iron oxide nanoparticles (SPIONs) and anticancer drugs DOX were coloaded into the thermal‐responsive liposomes for alternating magnetic field (AMF)‐triggered drug release (Figure 3A). Notably, a burst release of DOX from the liposomes was observed at 42°C compared with that at 37°C, exhibiting the thermal‐responsive behavior (Figure 3B).

It should be noted that in certain cases, thermal‐responsive materials could also be utilized for on‐demand drug release in response to endogenous hyperthermia in specific diseases treatment. For example, Wu et al.^123^ reported thermo‐sensitive polymeric micelles for febrile seizure therapy. These antiseizure drug‐encapsulated micelles could remain stable until the temperature was elevated to 39°C, which further trigger the micelle disassembly and following drug release. However, with the development of thermal‐responsive nanomaterials, there are still limitations that need to be solved, such as biocompatibility, stability, and particularly temperature responses at improved speed, sensitivity, and specificity.^119^ Hence, thermal‐responsive nanomaterials capable of more temperature‐sensitive, biocompatible, and biodegradable properties should be designed in the future.

### 2.5 Magnetic‐responsive nanomaterials

Magnetic field is a wireless and noninvasive approach for tissue penetration and without causing adverse effects to the body.^124^, ^125^ Magnetic nanomaterials are gaining popularity in biomedical applications, including diagnosis, drug delivery, and controlled release.^126^ Magnetic‐responsive nanomaterials are triggered by an external magnetic field and have drawn enormous attention in the field of nanomedicine. These nanomaterials can not only precisely control the drug‐loaded magnetic nanomaterials to the targeted site under the influence of the external magnetic field, but also serve as stimuli‐responsive factors for regulating drug release.^8^, ^23^, ^45^ The mechanisms of magnetic‐responsive nanomaterials can be classified into two categories: magnetic field‐induced hyperthermia and magnetic field‐guided drug targeting.^4^

Typically, magnetic‐responsive nanomaterials exhibit core–shell structures with commonly employed magnetic nanoparticles such as magnetite (Fe_3_O_4_) and maghemite (Fe_2_O_3_) serving as the magnetic core while being coated with inorganic, organic, or bio‐derived materials to form core–shell nanomaterials.^9^ For instance, biodegradable chitosan‐modified Fe_3_O_4_ nanoparticles (CS‐MNPs) were prepared by incorporating magnetic Fe_3_O_4_ in chitosan (a natural polymer with positive charge), which were then encapsulated with DOX as an antitumor drug.^46^ Under magnetic field, DOX–CS‐MNPs could aggregate, and quickly return to dispersed condition upon removal of external magnetic field, demonstrating that the DOX–CS‐MNPs are excellent magnetic‐responsive nanomaterials.

Interestingly, magnetic nanomaterials‐mediated local hyperthermia has emerged as a promising approach for cancer treatment.^44^ For example, Senturk et al.^45^ have constructed a radiofrequency–hyperthermia (RF–HT) system, which combined SPIONs with a natural compound curcumin, and modified it with a targeting ligand for GBM treatment (Figure 3C). The results showed that in the presence of RF‐field, the cell viability after GRGDS–Cur‐m‐PNPs treatment was significantly decreased owing to the effect of magnetic field‐mediated hyperthermia (Figure 3D). Interestingly, the magnetic field could also increase the cellular uptake.

Furthermore, magnetic‐responsive nanomaterials could also be performed as theranostic nanomaterials for magnetic resonance imaging (MRI). A polyethyleneimine (PEI)‐modified magnetic nanoparticle and a heparin‐coated superparamagnetic nanoparticle with a cationized protein β‐galactosidase have been developed by Chertok et al.^127^, ^128^ for drug delivery utilizing intracarotid administration. within comparison with intravenous injection, intracarotid administration of magnetic‐responsive nanomaterials exhibited high tumor‐targeting selectivity and could be utilized as MRI‐visible agents for both tumor therapy and diagnosis.

### 2.6 Light‐responsive nanomaterials

Due to their noninvasiveness and spatiotemporal precision, a variety of light‐responsive nanomaterials have been developed for achieving on‐demand drug release triggered by light with a specific wavelength in the ultraviolet, visible or near‐infrared (NIR) regions.^9^, ^129^ It has been reported that the conformational changes, chemical bond cleavage, or photothermal conversion of the light‐responsive materials under illumination could be the main mechanism for light‐triggered drug release.^24^

For instance, Zhong et al.^47^ have prepared an NIR‐responsive nanoparticle (cRGD–HN–DOX) by coating gold nanorods with PEG–b‐PCL–lipoic acid ester (PEG–PCL–LA) and modified with cRGD as a cell‐targeting peptide for GBM treatment (Figure 3E). In vitro drug release study displayed that the loaded DOX release from cRGD–HN–DOX was less than 10% in 4 h, illustrating its high stability. However, cumulative release of DOX was significantly boosted from 2.0 to 32.6% within 1 h upon NIR irradiation for 5 min (Figure 3F). With repeated laser treatment, DOX release exhibited NIR‐triggered drug release at 2, 3, and 4 h, demonstrating the DOX release from cRGD–HN–DOX could be remotely controlled by NIR laser irradiation. Besides, an enhanced DOX release could effectively enhance the antitumor activity on U87MG cells with cRGD–HN–DOX treatment, further supporting that the drug release from nanoparticles inside cells was greatly enhanced by NIR laser irradiation.

Nevertheless, the clinical applications of light‐responsive nanomaterials are largely limited by the poor tissue‐penetration abilities of light, especially for ultraviolet and visible light.

### 2.7 Ultrasound‐responsive nanomaterials

Compared with light‐responsive nanomaterials, ultrasound stimulation is capable of deeper tissue‐penetration capability, rendering it an attractive option for clinical applications. As a noninvasive and local stimulus, ultrasound can not only weaken the tight junctions of physiologic barrier (such as blood–brain barrier [BBB]) for a short time, but also control the drug release at pathological site, thus preventing toxicity to healthy tissues.^130^, ^131^ Hence, ultrasound‐responsive nanomaterials have also been applied for enhancing drug delivery, including gold nanoparticles, titanium dioxide nanosticks, barium titanate nanoparticles, and nanobubbles.^132^, ^133^, ^134^, ^135^ There are two mechanisms involved in ultrasound of controlled release, including thermal‐ and mechanical‐induction, which are mediated via radiation forces or cavitation phenomena, respectively.^21^

Ultrasound, a mechanical wave with thermal effects, has been utilized as a hyperthermia mediated method for improving the antitumor effect in glioma treatment.^136^ Kim et al.^48^ designed a closed‐loop trans‐skull MRI‐guided focused ultrasound (MRgFUS)–hyperthermia system via encapsulating DOX into thermosensitive liposomes (TSL–DOX) (Figure 3G). Upon MRgFUS irradiation, the fluorescence of DOX in the plasma was lower as compared with non‐FUS group. In addition, cellular uptake and penetration of DOX in the vessel were significantly enhanced when applied with mild hyperthermia of 41.5°C for 10 min. Both above results demonstrated that MRgFUS could efficiently control DOX release from thermosensitive liposomes to improve drug delivery in tumor site (Figure 3H).

Owing to the safety, convenience, and noninvasiveness, ultrasound‐responsive nanomaterials display unique advantages for real‐time diagnosis and treatment in clinic. However, it should be noted that some fundamental issues such as the tolerance range of ultrasound intensity and exposure time on human body should also be addressed when preparing the smart materials.

### 2.8 Multiple stimuli‐responsive nanomaterials

Due to the complexity of pathological microenvironment, multiple stimuli‐responsive nanomaterials have been engineered to be responsive to multiple triggers rather than a single stimulus to further improve the therapeutic efficacy for disease therapy. Hence, multiple stimuli‐responsive nanomaterials were designed to achieve on‐demand drug release and precise treatment.

By combining multiple responses of endogenous stimuli including lower pH value, overexpressed ROS, and GSH of pathological conditions, the design of pH/ROS or pH/GSH dual responsive nanomaterials have been applied to develop stimuli‐responsive nanomaterials. Sabourian et al.^49^ reported chitosan/HA nanocarriers loaded with quercetin, curcumin, and nerve growth factor protein that were responsive to both pH and ROS. Functionalized with TK diacid, a ROS‐responsive molecule, chitosan exhibited pH and ROS dual‐responsive property. In the presence of H_2_O_2_ and pH stimuli, the drug‐loaded nanomaterials demonstrated pH/ROS‐responsive release capability measured by both hydrodynamic diameter and drug cumulative release. Without H_2_O_2_, quercetin release from the nanomaterials was only about 20% over 48 h. However, the cumulative release accelerated up to 70% after H_2_O_2_ treatment (100 μM). A similar release behavior was observed when stimulated with decreased pH. The cumulative release of quercetin was only 19% at pH 7.25, whereas highly increased to 90% at pH 6.14 after 48 h, indicating these nanomaterials were comparatively stable in normal environment, while being capable of triggering drug release in a dual‐responsive manner governed by both pH and ROS stimuli. Similarly, Song et al.^50^ produced pH/GSH dual‐responsive nanogels by utilizing carboxymethyl chitosan nanogels modified with ANG as targeting peptide for DOX delivery, achieving a drug loading efficiency of 12.7% (Figure 3I,J). There was no sudden release effect at pH 7.4, whereas 84.2% DOX was released from nanogels at pH 5.5 within 36 h in the presence of GSH (10 mM), demonstrating that this nanogel has excellent pH and GSH dual‐responsive properties (Figure 3K).

Furthermore, combining endogenous and exogenous stimulus have also been demonstrated as a useful therapy of cancer treatment. For instance, Cao et al.^51^ synthesized an intelligent nanoplatform loaded with temozolomide (TMZ), and functionalized with HA as a gatekeeper on the surface to prevent premature drug leakage. In normal environment, TMZ release is less than 10% over a period of 14 h. With the assistance of hyaluronidase, an obvious release of TMZ was presented, and up to 36% in the acid tumor environment. Interestingly, with 808‐nm laser irradiation, the cumulative release of TMZ was significantly increased to 70%. Taking together, this nanoplatform processed pH, enzyme and light triple‐responsive properties, facilitating its further application and reducing side effects.

In general, multiple stimuli‐responsive nanomaterials integrate the advantages of various responses, resulting in more complex design requirement and necessitating further evaluation of their multiple response properties.

## 3 BIOMEDICAL APPLICATIONS

Given that stimuli‐responsive nanomaterials are highly sensitive to endogenous and exogenous stimulus, they are regarded as ideal candidates for biomedicine. In this section, we will discuss the biomedical applications of stimuli‐responsive nanomaterials, including CVDs, cancer, neurological disorders, inflammation, bacterial infection, and so on (Table 2).

**TABLE 2:** Stimuli‐responsive materials for biomedical applications.

Biomedical applications	Disease	Stimuli	Nanomaterials	Applications	References
CVDs	AS (ApoE−/−)	ROS	TPTS/C/T	Drug delivery	137
CVDs	AS (ApoE−/−)	pH or ROS	RAP/Ac‐bCD NP, RAP/Ox‐bCD NP	Drug delivery	138
CVDs	AS (ApoE−/−)	ROS	HA–Fc/NP^3^_ST_	Drug delivery	139
CVDs	AS (ApoE−/−)	US	CDNPs	Imaging (US and NIR imaging)	140
CVDs	AS (ApoE−/−)	pH	MeOTTI–PMEA NPs	Imaging (fluorescence imaging)	141
CVDs	AS (ApoE−/−)	ROS	R‐Lipo@HDB/CH1055	Imaging (fluorescence imaging)	142
CVDs	AS (ApoE−/−)	ROS	TPAMCF	Imaging (fluorescence imaging)	143
CVDs	AS (ApoE−/−)	LDs and HClO	iSHERLOCK	Imaging (fluorescence imaging)	144
CVDs	IS (photothrombosis method in mice)	ROS	IR–LnNPs	Imaging (NIR‐II luminescence imaging)	145
CVDs	Cerebral ischemic area (MCAO)	pH	Fe_3_O_4_‐loaded mPEG‐P(DE‐DPA)LG micelles	Imaging (MRI)	146
CVDs	IS (tMCAO)	pH	RAPA/Gd^3+^@NPs	Theranostics (MRI, fluorescence imaging, neuroprotective effects)	147
CVDs	AS (ApoE−/−)	MMP‐9/ROS	PLCDP@PMH	Theranostics (PA, lipid removal, anti‐inflammatory, enhanced lipid efflux)	148
CVDs	AS (ApoE−/−)	pH	MMNS–CS–DS	Theranostics (MRI, anti‐inflammatory, lipid‐regulating, and autophagy)	149
CVDs	AS (ApoE−/−)	ROS	LFP/PCDPD	Theranostics (fluorescence imaging, anti‐inflammatory, lipid removal)	150
CVDs	AS (FeCl_3_ and ApoE−/−)	ROS	Fe_3_O_4_@SiO_2_–CDs	Theranostics (fluorescence imaging, MRI, inhibition the formation of plaques)	151
CVDs	AS (ApoE−/−)	ROS	RBC/LFP@PMMP	Theranostics (fluorescence imaging, anti‐inflammation)	152
Cancer	Breast cancer	pH/NIR	MEL/Cypate@HA	Drug delivery	153
Cancer	Liver tumor	Redox/pH	DOX loaded supramolecular NPs	Drug delivery	154
Cancer	GBM	pH	Ang–RBCm–CA/siRNA	Drug delivery	28
Cancer	Glioma	GSH	AuNWs	Imaging (MRI, PA)	155
Cancer	Glioma	GGT	NRh‐G‐NPs	Imaging (fluorescence imaging)	156
Cancer	Colorectal cancer	ROS	IONPs–ICG–HA	Imaging (PA, photothermal imaging, fluorescence imaging)	157
Cancer	Breast cancer	pH	H‐MnO_2_–PEG/C&D	Theranostics (MRI, immunotherapy)	158
Cancer	Triple‐negative breast cancer	pH/GSH/Glucose	BDS–GO*_x_* @MnO*_x_*	Theranostics (CT, MRI, starvation therapy and CDT)	159
Cancer	Colonic cancer	pH/ROS	AtkCPTNPs	Theranostics (MRI, chemotherapy, PDT)	160
Neurological disorders	Epilepsy	Electric	ANG–PHT–ERHNPs	Drug delivery	161
Neurological disorders	Epilepsy	Electric	PPY–PDA–PHT–ANG	Drug delivery	162
Neurological disorders	AD	ROS	Ab–PEG–LysB/CUR	Drug delivery	163
Neurological disorders	AD	Cu^2+^	RPS1	Imaging (PA)	164
Neurological disorders	AD	Aβ	DMP	Imaging (NIR‐II fluorescence imaging)	165
Neurological disorders	Glioma	pH/H_2_O_2_	iRPPA@TMZ/MnO	Theranostics (MRI, chemotherapy)	166
Neurological disorders	Epilepsy	Electric	TFP@cargo	Theranostics (fluorescence imaging, chemotherapy)	167
Inflammation	IBD (DSS‐induced colitis model)	pH/Redox	BDS–ATP–CMI	Drug delivery	168
Inflammation	Periodontitis	pH	Chitosan‐based hydrogel loaded with PTB	Drug delivery	169
Inflammation	Hepatic inflammation	H_2_S	1‐PEI‐DCNPs	Imaging (NIR‐II fluorescence imaging)	170
Inflammation	Acute pancreatitis	pH	CRCS	Imaging (fluorescence imaging, MRI)	171
Inflammation	OA	pH/MMP‐13	MRC–PPL@PSO	Theranostics (anti‐inflammatory, fluorescence imaging)	172
Inflammation	Arthritis and AS	ROS	TPP@PMM	Theranostics (two‐photon imaging, anti‐inflammatory)	173
Bacterial infection	*S. aureus* and *E. coli*	pH	PE‐*g*‐pAPDMAPA	Drug delivery	174
Bacterial infection	MRSA biofilms	pH	ICG–ZnS NPs	Drug delivery	175
Bacterial infection	*S. aureus*	Hyaluronidase	HA–CP@Fe_3_O_4_	Drug delivery	176
Bacterial infection	*S. aureus*	pH/H_2_O_2_	PT	Drug delivery	177
Bacterial infection	*S. aureus* and *E. coli*	pH	AgNCs	Drug delivery	178
Bacterial infection	Musculoskeletal infections	Magnetic	Fe_3_O_4_ MNP‐loaded chitosan ross‐linked with PEGDMA	Drug delivery	179
Bacterial infection	*S. aureus*	pH	MDVG‐1	Imaging (MRI)	180
Bacterial infection	*S. aureus*	MMP‐2	MPD‐1	Imaging (MRI)	181
Bacterial infection	*E. coli* and *S. aureus*	pH	PPEGMA‐*b*‐P(DPA‐coHEMA)–Ce6	Theranostics (fluorescence imaging, antimicrobial)	182
Bacterial infection	MRSA	ROS	_D_‐AzAla@MIL‐100 (Fe) NPs and PS NPs	Theranostics (fluorescence imaging, PDT)	183
Bacterial infection	MRSA biofilm	pH	MnO_2_–BSA/PEG–Ce6 NSs	Theranostics (fluorescence, MRI, PDT)	184
Bacterial infection	MRSA	Hyaluronidase	MoS_2_@HA–Ce6	Theranostics (fluorescence imaging, PTT, PDT)	185
Bacterial infection	MRSA	pH/ROS/Hyaluronidase	PLNPs@MSN@CA–HA–MnO_2_	Theranostics (persistent luminescence imaging, CDT)	186
Bacterial infection	MRSA	pH	PLNP@PANI–GCS	Theranostics (persistent luminescence imaging, PTT)	187

### 3.1 Cardiovascular diseases

CVDs, including atherosclerosis (AS), stroke, and myocardial infarction, are the leading cause of human death worldwide due to the high incidence.^188^ Conventional treatments for CVDs include surgery and systematic medications.^189^, ^190^ But the therapeutic outcomes are largely hampered by short half‐life, low bioavailability, and nonspecific distribution of the drugs. In the face of such shortages, nanomaterials, capable of targeted drug delivery, bioimaging, and enhanced theranostics, have been emerged as an effective and sensitive control technology to provide personalized medication in the treatment of CVDs.^191^, ^192^ Besides, the pathological microenvironment of CVDs characterized by acidic pH, elevated ROS levels, activated enzymes, and inflammation factors can be utilized as endogenous biomarkers to design stimuli‐responsive nanomaterials for on‐demand drug delivery, imaging and theranostics.^193^

#### 3.1.1 Drug delivery in CVDs

Drug delivery systems are considered as a technology using a number of functionalized nanomaterials to precisely target pathogenic sites.^194^ Stimuli‐responsive nanomaterials have gained great progress for designing controllable drug delivery systems, owing to their excellent spatiotemporal and controllable capabilities. Following administration, stimuli‐responsive drug delivery systems will passively or actively target to diseased sites. Once triggered by endogenous or exogenous stimulus, the chemical or physical structural changes of these nanomaterials may induce on‐demand drug release, thereby improving the efficiency of drug delivery.^23^ Consequently, the utilization of stimuli‐responsive nanomaterials in drug delivery of therapeutics is a promising approach for diseases treatment.

**FIGURE 4:**
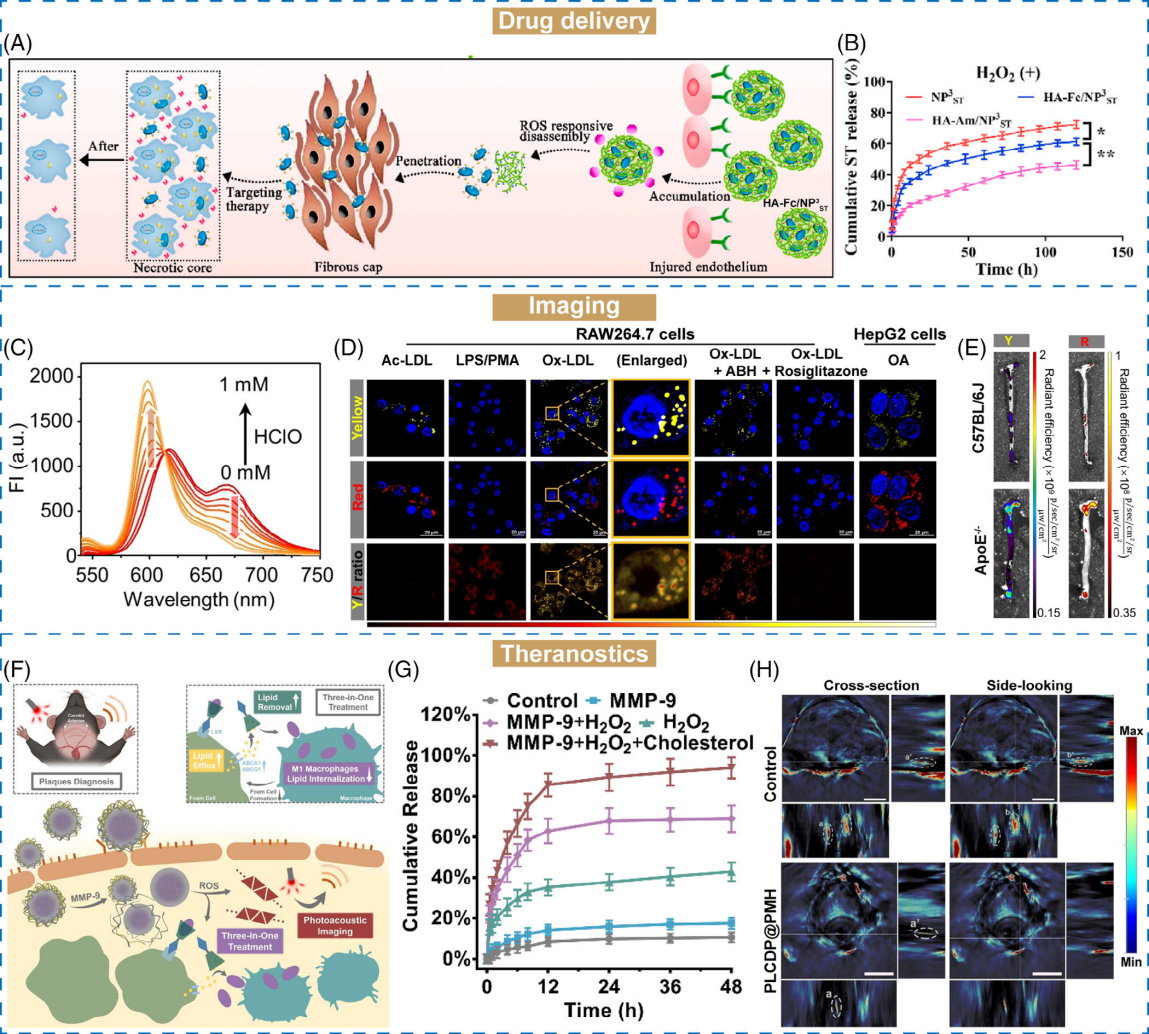
The biomedical applications of stimuli‐responsive nanomaterials in CVDs. (A) Schematic illustration of the preparation of ROS‐responsive HA–Fc/NP^3^_ST_ nanomaterials and in vivo behavior. (B) Drug release profiles of HA–Fc/NP^3^_ST_ with H_2_O_2_ incubation. (A and B) Reproduced with permission from He et al.^139^ Copyright 2023, Elsevier BV. (C) Fluorescence spectra of MTB‐B‐CF_3_ after HClO treatment. (D) Fluorescent images of RAW264.7 cells and HepG2 cells after various stimulants with MTB‐B‐CF_3_. (E) Ex vivo imaging of aortas in C57BL/6J and ApoE^−/−^ mice after treatment with MTB‐B‐CF_3_‐incorporated Ac‐LDL. (C–E) Reproduced with permission from Ye et al.^144^ Copyright 2022, Wiley VCH. (F) Schematic illustration of the in vivo process of PLCDP@PMH for AS theranostics. (G) In vitro Pred release manner of PLCDP@PMH. (H) Typical cross‐section and side‐looking images of AS plaques in carotid arteries with or without PLCDP@PMH treatment. (F–H) Reproduced with permission from Ma et al.^148^ Copyright 2023, Wiley VCH.

AS is the central inflammatory disease of CVDs, which caused by the accumulation of lipids on the wall of blood vessels, followed by thickening and clogging.^195^, ^196^ A series of stimuli‐responsive nanomaterials have emerged as crucial tools for targeted drug delivery and controlled release in AS treatment.^137^, ^138^, ^197^, ^198^, ^199^, ^200^ He et al.^139^ proposed ROS‐responsive nanoassemblies comprising β‐cyclodextrin (β‐CD)‐anchored discoidal recombinant high‐density lipoprotein (NP^3^_ST_) with HA–ferrocene (Fc) conjugation (HA–Fc/NP^3^_ST_). Under the excessive ROS environment of AS, the hydrophobic Fc would be oxidized into hydrophilic ferrocenium ion, resulting in the disintegration of HA–Fc/NP^3^_ST_ and NP^3^_ST_ release (Figure 4A). In this study, we investigated the oxidation properties of HA–Fc/NP^3^_ST_. Nonresponsive nanoassemblies based on HA–amantadine (Am)/NP^3^_ST_ were constructed as the unresponsive control. Upon labeling with fluorescence dyes (Rho123–HA–Fc/NP^3^_ST_–RITC), the emission intensity of Rho123 was decreased, and that of RITC was increased, suggesting fluorescence resonance energy transfer in the presence of ROS (1 mM H_2_O_2_). However, for Rho123–HA–Am/NP^3^_ST_–RITC, the emission intensity of either Rho123 or RITC showed no obvious changes after ROS intervention. Subsequently, the ROS‐responsive drug release of HA–Fc/NP^3^_ST_ was investigated. About 55% of NP^3^_ST_ was released from HA–Fc/NP^3^_ST_ after 72 h incubation with ROS, whereas only 38.2% in the absence of ROS (Figure 4B). Conversely, the drug release profiles of HA–Am/NP^3^_ST_ exhibited no discernible differences with or without ROS intervention, further confirming that HA–Fc was an excellent ROS‐responsive nanomaterial for targeted drug delivery in AS therapy.

Specifically, owing to the narrowing blood vascular in AS, the wall shear stress in the plaques (31.9–136.09 dyn cm^−2^) was higher than that in normal vessels (1–10 dyn cm^−2^).^201^ Hence, this enhanced shear stress presents opportunities for designing shear‐stress‐responsive nanomaterials for AS treatment.^202^, ^203^ Shear‐stress‐sensitive lenticular vesicles of an artificial 1,2‐diaminophospholipid were constructed by Holme et al.^204^ for targeted drug delivery to AS plaques. The designed Pad–PC–Pad vesicles were stable under normal stress but could break down and release their loading cargos when subjected to higher shear‐stress levels. This shear‐stress‐responsive changes could possibly be attributed to the lenticular morphologies of Pad–PC–Pad, leading to their instability along equator. However, the hemodynamic variations are influenced by factors such as the blood circulatory system, blood vessel bifurcations and arterial openings, which enquired that the shear‐stress force should be sensitive enough to fight against these interfering factors and precisely control the drug release.^205^ From this point of view, shear‐stress stimulus could be combined with other stimulus such as overexpressed ROS, realizing dual‐responsive drug release and improving the therapeutic efficiency against AS.^206^

#### 3.1.2 Imaging in CVDs

Owing to the slow progression and mild symptoms of CVDs, it is difficult to diagnose the pathological changes in time.^207^ In recent years, computed tomography (CT), MRI, fluorescence imaging, ultrasonography (US), and so on have been utilized as a real‐time, accurate, and continuous auxiliary for CVDs diagnosis.^140^, ^208^, ^209^, ^210^ Numerous stimuli‐responsive approaches have been developed for imaging AS plaques on the basis of their pathology like lipid droplets (LDs) and unregulated ROS levels,^141^, ^142^, ^143^ which are considered as two hallmarks of AS. LDs in foam cells within AS lesions are formed by the triggering of oxidized low‐density lipoprotein.^211^ Labeling LDs with fluorescence probes can help us to detect the AS plaques.^212^ Ye et al.^144^ proposed a three‐in‐one fluorescent probe (iSHERLOCK) capable with dual‐target sequentially activated properties for precise detection of AS plaques. LDs and hypochlorous acid (HClO, excessively produced in inflammatory diseases) were used as respective indicators for plaques detection. The iSHERLOCK probe MTB‐B‐CF_3_ was prepared by combining LDs‐responsive tracker (BODIPY) and HClO‐responsive ligand (MTB). In the presence of LDs, MTB‐B‐CF_3_ showed about 160‐fold fluorescence enhancement. In contrast, there was no obvious fluorescence change in biological conditions. Likewise, upon addition of HClO, the maximal emission band of MTB‐B‐CF_3_‐LD mimetic (MTB‐B‐CF_3_‐LDM) shifted from 615 to 600 nm with increasing concentration of HClO (Figure 4C). It is worth mentioning that these processes could be completed within a few minutes. In vitro cells studies demonstrated that MTB‐B‐CF_3_ was colocated with the LDs and HClO distribution in foam cells (Figure 4D). In the ApoE^−/−^, increased fluorescence signal was observed in the AS plaques, further suggesting the potential application of iSHERLOCK for precise detection of AS lesions (Figure 4E).

It has been reported that approximately 90% of strokes are ischemic and caused by AS, and continuous ischemia will result in serious injury when untreated timely.^199^ IS accounts for 85% of all strokes, which is caused by the abruption of cerebral perfusion.^213^ When ischemia occurs, the pH at cerebral site decreased to 5.9, whereas the pH of ischemic core and peri‐ischemia penumbra ranges from 6.0 to 6.9.^214^ Besides, ROS is another biomarker in IS processes with altered expression levels.^145^ The variations in pH and ROS levels between pathological and normal sites give an opportunity for designing pH and ROS‐responsive nanomaterials for IS treatment.^145^, ^147^ Based on these characterizations of the IS microenvironment, Yang and coauthors^146^ proposed a pH‐responsive micelle encapsulated with Fe_3_O_4_ nanoparticles for the detection of cerebral ischemic areas. Ischemic tissues are more acidic pH than physiological tissues owing to the accumulation of lactic acid, thereby offering potential for the development of pH‐responsive nanomaterials for sensitive imaging. After intravenous injection of Fe_3_O_4_‐loaded mPEG‐P(DPA‐DE)LG micelles into the mice with middle cerebral artery occlusion (MCAO) pretreatment, the T_2_‐weighted MRI of ischemic section was performed on the ischemic section. The results showed that an obviously decreased signal was caught with pH‐responsive micelles administration, which caused by the released Fe_3_O_4_ following pH‐sensitive demicellization. Conversely, no signal decreases were observed in the nonresponsive group, proving the potentiality of pH‐active MR diagnostic imaging in ischemic diseases.

#### 3.1.3 Theranostics in CVDs

As CVDs progress and deteriorate, it could cause serious damages to life quality of patients. Although clinical imaging approaches provide moderate information on the severity of vascular stenosis, identifying high‐risk lesions associated with CVDs remains challenging.^215^ Hence, accurate diagnosis and effective therapy are important for risk stratification and reducing mortality rates related to CVDs.^148^ Stimuli‐responsive nanomaterials have garnered considerable attention in the field of the theranostics for CVDs, especially in AS, due to their on‐demand release, lesion‐targeting specificity, and excellent bioavailability.^205^, ^216^ After combination with imaging toolbox, these nanomaterials could precisely detect the pathological condition, as well as monitoring the drug targeting efficiency and accumulation.

β‐CD serves as a lipid solubilizer for lipid removal of AS.^217^ Ma et al.^148^ constructed an MMP‐9/ROS dual‐responsive therapeutic complex (PLCDP@PMH) based on β‐CD for both imaging and treatment of AS (Figure 4F). Liver X receptor ligand T0901317 was bridged with β‐CD, and glucocorticoid prednisolone (Pred) was loaded into β‐CD via host–guest interaction. After that, this LCDP complex was encapsulated with a photoacoustic imaging (PA) probe (PMeDTDPP‐EDOT), followed by coating of oxidized HA, ROS‐responsive polymer PMEMA and MMP‐9 sensitive peptide for targeting and on‐demand release in AS lesions. In vitro studies demonstrated that over 92% of Pred was released after 48 h, suggesting the excellent MMP‐9/ROS dual‐responsive ability of PLCDP@PMH (Figure 4G). Upon administration, PLCDP@PMH actively accumulated in the AS lesions based on the interactions between HA and overexpressed CD44 on endothelium cells. The overexpressed ROS and MMP‐9 within plaques would trigger the breakage, disintegration, and subsequent release of cargos from PLCDP@PMH. In ApoE^−/−^ model, PA signals of the plaques were stronger than normal tissues, and the plaques formation and progression were efficiently inhibited, realizing in vivo diagnosis, plaque‐formation inhibition and lipid removal for AS theranostics (Figure 4H).

In another study, Cheng and associates^147^ developed a pH‐responsive theranostic nanoplatform for the delivery of rapamycin (RAPA) in IS treatment. Ce6‐labeled pH‐sensitive segments (PDPA) self‐assembled with RAPA and Gd^3+^ to form RAPA/Gd^3+^@NPs for dual‐modal imaging including MRI and NIR fluorescence imaging. These nanomaterials were stable and self‐quenched at physiological condition. Under pathological condition at pH 6.0, over 80% of RAPA was released within 4 h, revealing the pH‐responsive property of RAPA/Gd^3+^@NPs. On the contrary, only 20% of Gd^3+^ was released within 24 h, which is mainly due to the strong chelation between Gd^3+^ and Ce6 molecules. In vitro MRI and fluorescence imaging study showed that the longitudinal correlation coefficient and fluorescence intensity of RAPA/Gd^3+^@NPs at pH 6.0 were higher than that at pH 7.4, demonstrating the acid‐activated MR and fluorescence behavior of RAPA/Gd^3+^@NPs. Moreover, in vivo MR and fluorescence imaging were performed on transient MCAO rat models. The signal and fluorescence intensity in the right hemisphere of cerebral of mice injected with RAPA/Gd^3+^@NPs increased, indicating the efficient accumulation of these nanomaterials in the ischemic region. Besides, the released RAPA exerted excellent neuroprotective effects for IS treatment. Therefore, the use of RAPA/Gd^3+^@NPs nanomaterials offer a promising approach to tackle IS by theranostics.

Collectively, stimuli‐responsive nanomaterials have demonstrated distinct advantages in drug delivery, imaging, and theranostics for CVDs by responding to both endogenous and exogenous stimulus. Especially, abnormal shear stress in AS gives potential of constructing shear‐stress‐responsive nanomaterials. However, the therapeutic outcomes of these nanomaterials may be further hindered by their sensitivity toward the diseased microenvironment, and discrepancies between animal models used for testing and human.

### 3.2 Cancer

Cancer, which is a social problem in the 21st century, results in 9.7 million deaths worldwide in 2022 and is considered as the second leading cause of mortality after CVDs.^218^, ^219^ Clinical approaches for cancer treatments include surgical resection, chemotherapy, and radiotherapy. Chemotherapeutics have been widely used for a long time to cancer treatment, but their therapeutic effects are largely hampered by potential side effects, such as drug resistance, short half‐life, systemic toxicity, and the intricate biology of cancer.^220^, ^221^ Compared with normal tissues, TME is characterized by acidic pH values, high GSH and ROS expression, overexpressed enzymes, hypoxia, and so on, which gives the opportunities for designing stimuli‐responsive nanomaterials to achieve on‐demand drug release, reduce side effects, and improve efficacies.^222^, ^223^ Recently, a variety of TME‐responsive nanomaterials have been widely developed and used in biomedical applications, such as drug delivery, imaging, and theranostics.^224^

#### 3.2.1 Drug delivery in cancer

**FIGURE 5:**
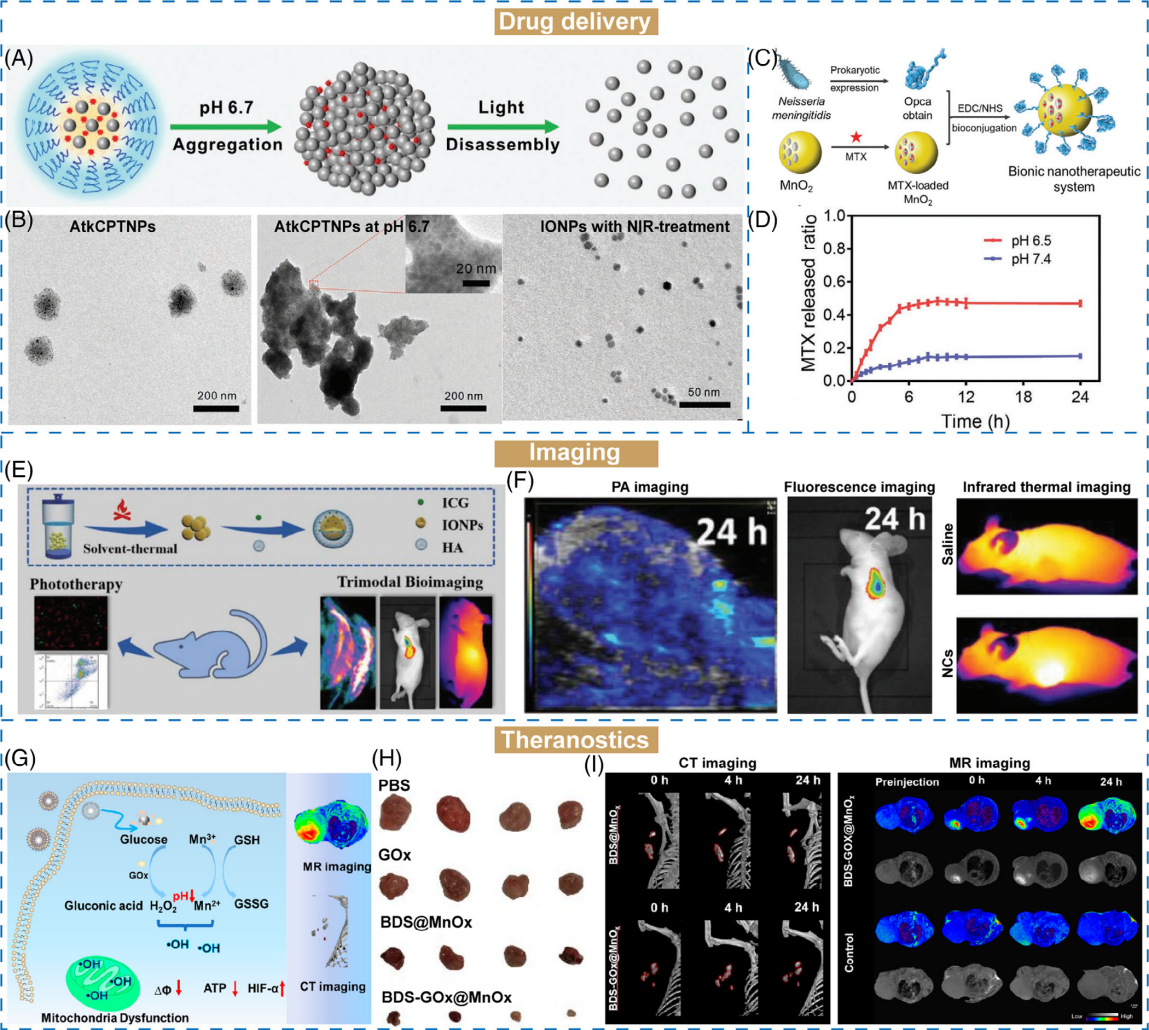
The biomedical applications of stimuli‐responsive nanomaterials in cancer. (A) Schematic illustration of pH‐induced aggregation and ROS‐triggered degradation of AtkCPTNPs. (B) TEM imaging of AtkCPTNPs before or after treated with acid pH or light. (A and B) Reproduced with permission from Cheng et al.^160^ Copyright 2021, Wiley VCH. (C) Schematic illustration of the preparation of MTX@MnO_2_–Opca nanomaterials. (D) The release profiles of MTX at pH 7.4 or 6.5. (C and D) Reproduced with permission from Dong et al.^107^ Copyright 2022, Wiley VCH. (E) Schematic illustration of the synthesis of IONPs–ICG–HA. (F) In vivo PA, fluorescence and infrared thermal imaging after IONPs–ICG–HA injection. (E and F) Reproduced with permission from Wang et al.^157^ Copyright 2019, Wiley VCH. (G) Schematic illustration of the antitumor effects and imaging of BDS–GO*_x_* @MnO*_x_* NPs. (H) Digital images of tumors. (I) CT and MR imaging of mice after BDS @MnO*_x_* or BDS–GO*_x_* @MnO*_x_* treatment. (G–I) Reproduced with permission from Li et al.^159^ Copyright 2023, American Chemical Society.

Owing to the complexity of TME, a series of stimuli‐responsive nanomaterials have been developed for drug delivery. Size‐changeable strategy is designed for delivering drugs to achieve effective tumor targeting, accumulation and retention at tumor sites. The changeable size of these nanomaterials with either size‐shrinkable or size‐increasing property is mainly relied on their structural properties to respond to endogenous and exogenous stimulus.^225^ Cheng et al.^160^ reported a size‐changeable nanotheranostic agent (AtkCPTNPs) using polyprodrug‐modified iron oxide nanoparticles (IONPs), which was prepared by assembly of pH‐responsive polymer, ROS‐responsive prodrug‐modified IONPs, and a photosensitizer (Figure 5A). After passively targeting to the tumor site, this nanomaterial was aggregated under acidic environment (size growth from 90 to 300 nm). Then, the aggregates were transformed into small‐sized IONPs under the stimuli effects of produced ROS with white light irradiation (size reduction to 17 nm) (Figure 5B). The programmed size changes could effectively enhance tumor retention and drug release and decrease the toxicity of nanotheranostic agent via accelerated elimination.

Stimuli‐responsive nanocarriers have been extensively employed for drug delivery for cancer treatment. Methotrexate (MTX) is limited in poor solubility and weak targeting ability in clinical cancer treatment. To address these challenges, Dong et al.^107^ proposed a hollow MnO_2_ nanoparticles loaded with chemotherapeutic drug MTX. Opca, an outer membrane invasion protein for brain targeting, was modified on the surface of MnO_2_ nanoparticles to form a bionic nanotherapeutic system (MTX@MnO_2_–Opca) (Figure 5C). In TME‐mimicking environment (pH 6.5), about 50% of MTX was released within 12 h, which was higher than that at pH 7.4 of 15% (Figure 5D). Besides, the MTX@MnO_2_–Opca was catalyzed by H_2_O_2_ to produce O_2_, confirming the effective H_2_O_2_‐responsive ability of MnO_2_. Therefore, MnO_2_ nanocarriers exhibited good drug encapsulation, degradability, and TME‐responsive properties, which could be further used as a potential nanomaterial for advanced drug delivery applications.

#### 3.2.2 Imaging in cancer

Medical imaging technology is an associated strategy in clinical diagnosis and treatment, enabling real‐time monitoring of the biological process of cancer and drug efficiency.^18^, ^226^, ^227^ Currently, diverse diagnostic modalities including optical imaging, MRI, and PA, have been proved their effectiveness in cancer imaging.

Optical imaging is commonly used imaging technique based on fluorescent agents, owing to its simplicity, high sensitivity, and intuitive results. But the strong and nonspecific binding with plasma proteins, nonspecific targeting, and unsatisfactory photostability of fluorescent agents hinder the broader their applications.^228^, ^229^ To solve these challenges, stimuli‐responsive nanomaterials can improve the in vivo tumor‐specific targeting ability and pharmacodynamics of fluorescent agents for cancer imaging. A recent study reported the use of γ‐glutamyl traspeptidase (GGT) as a biomarker to develop GGT‐responsive NIR nanomaterials (NRh‐G‐NPs), by conjugating GGT‐specific substrate γ‐glutamic acid (γ‐Glu) with cyanine fluorophore (NRh‐NH_2_), which enables switchable fluorescence imaging and glioma therapy.^156^ Triggered by the overexpressed GGT in the TME, nonfluorescent NRh‐G‐NPs were cut off to form NRh‐NH_2_‐NPs and showed a strong fluorescence along with photothermal ability, suitable for both cancer diagnosis and photothermal therapy (PTT). This strategy offers potential benefits such as reduced toxicity toward normal tissues during the blood circulation. It was anticipated to improve the therapeutic efficiency to tumor, additionally facilitating on‐demand fluorescence imaging.

Wang et al.^157^ developed an H_2_O_2_‐responsive nanoplatform derived from IONPs (IONPs–ICG–HA), which was decorated with ICG and HA via electrostatic interactions for multimodal imaging‐guided cancer therapy (Figure 5E). In this nanoplatform, the combination of ICG and IONPs could not only improve the photostability of ICG, but also enhance the antitumor effect on H_2_O_2_‐responsive triggering Fenton‐reaction. Under upregulated H_2_O_2_ condition, IONPs exhibited improved catalytic activity to produce ∙OH. After administration for 4 h, both PA and fluorescence signals enhanced over time (Figure 5F). Besides, IONPs–ICG–HA owned superior light‐to‐heat conversion ability under laser irradiation, enabling in‐depth imaging. The results showed that the temperature of tumor sites could reach a temperature of 50.9°C within 5 min under irradiation and exhibited excellent photothermal efficacy in vitro and in vivo experiments. This study presents a novel strategy for designing stimuli‐responsive nanomaterials functionalized with PA/infrared thermal/fluorescence imaging.

#### 3.2.3 Theranostics in cancer

As a novel technology in clinical cancer therapy, theranostics integrates diagnosis and therapeutic abilities into one system, generating real‐time signals to promote the therapeutic processes of cancer treatment.^230^ Early diagnosis, timely therapy, and real‐time monitoring are crucial in the fight against cancer. It has been reported that TME‐responsive nanomaterials in theranostics could specifically amplify imaging signals, enhance drug accumulation at tumor sites, and promote deep penetration.^231^, ^232^

CDs are enzymatically degraded products possessing highly branched hydroxyl moieties and hydrophobic cavities. As a building block, CDs can be used in the preparation of stimuli‐responsive CD‐based nanomaterials through incorporating ideal responsive functionalities. Stimuli‐responsive CD‐based nanoplatforms for cancer theranostics, including pH‐responsive, redox‐responsive, enzyme‐responsive, and so on, have been summarized by Yao and associates.^233^ Through stimuli‐responsive design, CD‐based nanoplatforms can provide sensitive and real‐time imaging of the biodistribution of drugs and realize imaging‐guided cancer treatment.

In another study, a pH/GSH/glucose‐responsive nanozyme was designed by preparing a bismuth‐manganese core–shell nanoflower loading with glucose oxide (BDS–GO*_x_* @MnO*_x_*) (Figure 5G).^159^ The MnO*_x_* shell could serve as a “smart switch” with endogenous microenvironment‐responsive properties, including glucose, H_2_O_2_, and GSH‐responsiveness. Under weak acidic environment at pH 6.5, about 80% of GO*_x_* was released within 48 h, but only 21% of GO*_x_* at pH 7.4. The similar acidic‐dependent release performance was observed in the Mn releasing profiles. As an effective catalytic enzyme, GO*_x_* could exhaust glucose in the TME to produce H_2_O_2_ via starvation therapy. Under glucose condition, the consumption of glucose gradually enhanced under pH‐activated effects at pH 6.5 compared with pH 7.4, confirming its efficacy. Besides, the H_2_O_2_ and GSH dual‐responsive properties of MnO*_x_* further enhanced the generation of ∙OH and exhaustion of GSH. Both in vitro and in vivo results suggested that BDS–GO*_x_* @MnO*_x_* nanozymes could enhance the antitumor effects (Figure 5H). Furthermore, owing to the high X‐ray attenuation coefficient of bismuth and longitudinal relaxivity of Mn, BDS–GO*_x_* @MnO*_x_* nanozymes could serve as a TME‐responsive agent for both CT and MRI imaging (Figure 5I). In conclusion, this study provided a potential strategy that is multiresponsive for cancer diagnosis and treatment.

Owing to the complex characteristics exhibited by TME, such as changes in pH levels, oxidative stress, and protein expression patterns, stimulus‐responsive nanomaterials have been widely applied in cancer therapy. However, there are a few challenges need to be addressed: (1) due to the tumor heterogeneity and mutative TME, used tumor models are drastically differ from patients with cancer; (2) the potential toxicity and long‐term biosafety of these nanomaterials are mainly relied on their targeting capabilities.

### 3.3 Neurological disorders

Neurological disorders are recognized as a leading medical and societal burden and disability in the world owing to the limitation of efficient treatments.^234^ Currently, prevalent neurological disorders, such as Alzheimer's disease (AD), Parkinson's disease (PD), epilepsy, and so forth, arise from genetic factors, aging, trauma, environmental factors, and/or altered lifestyle.^235^, ^236^ Conventional therapies for neurological disorders include surgery, chemotherapy, and physical therapy.^237^ However, nearly 98% of therapeutics is blocked from the brain by the BBB, a physical transport barrier that strictly regulates the import and export of substances into the brain.^238^ With assistance of nanotechnology, the drug accumulation at the pathological site is significantly enhanced, giving improved therapeutic outcomes and minimized side effects associated with therapeutics administration.^239^, ^240^, ^241^, ^242^ Moreover, stimuli‐responsive nanomaterials have been developed as versatile switching systems capable of spatially and temporally controlled drug release for precise drug delivery applications including imaging and theranostics of neurological disorders.^243^

#### 3.3.1 Drug delivery in neurological disorders

**FIGURE 6:**
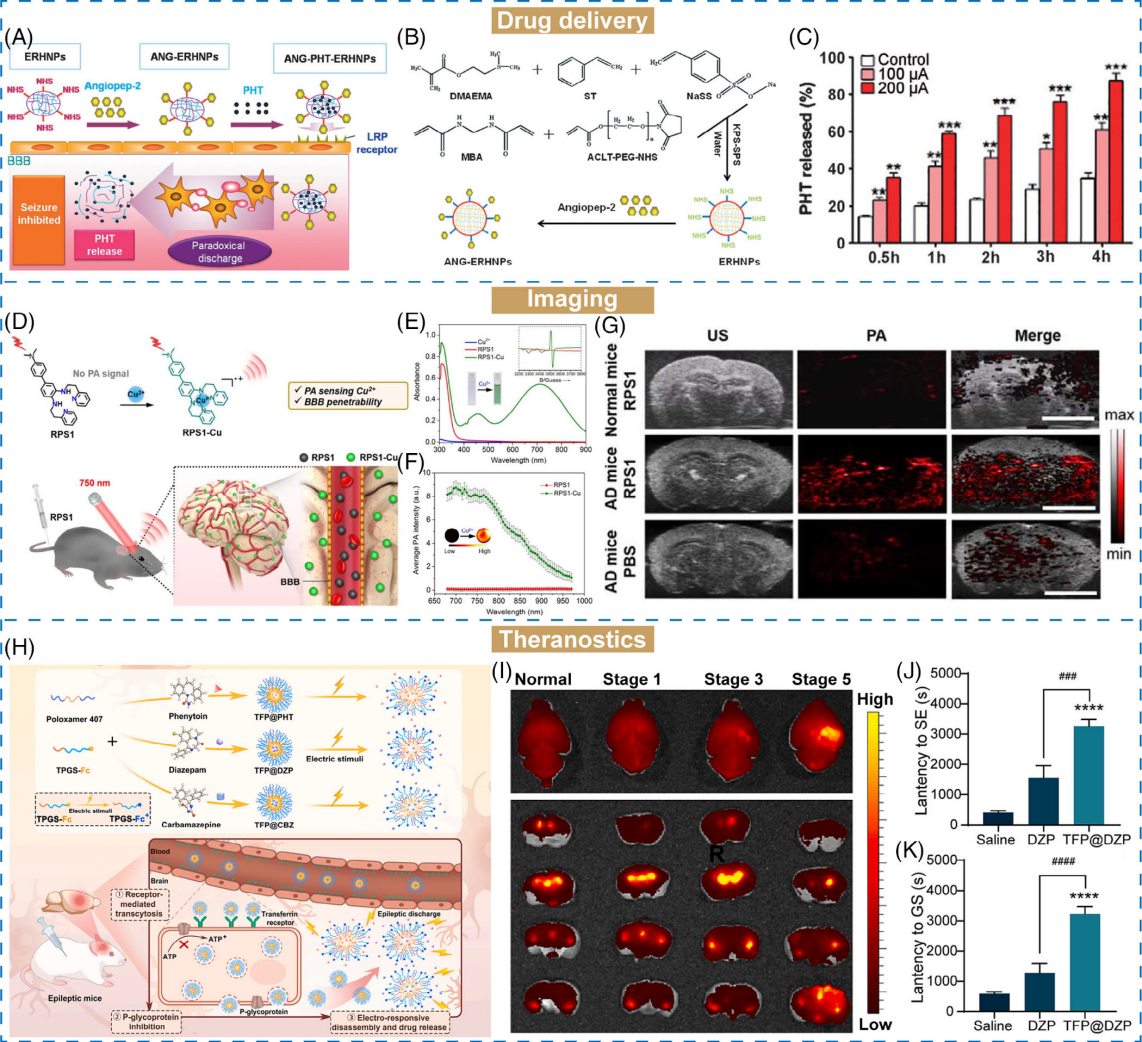
The biomedical applications of stimuli‐responsive nanomaterials in neurological disorders. (A) Schematic illustration of the preparation of electro‐responsive hydrogel nanoparticles. (B) The synthesis progress of the ANG–ERHNPs. (C) The release of PHT from ANG–ERHNPs triggered by an external electric field. (A–C) Reproduced with permission from Ying et al.^161^ Copyright 2014, Wiley‐VCH. (D) Schematic illustration of RPS1 detecting Cu^2+^ with PA imaging. (E) The absorption spectra of Cu^2+^, RPS1 and RPS1‐Cu. (F) The PA spectra of RPS1 and RPS1‐Cu. (G) PA images of normal and AD mice after RPS1 and PBS injection. (D–G) Reproduced with permission from Wang et al.^164^ Copyright 2019, Wiley‐VCH. (H) Schematic illustration of the synthesis of electro‐responsive TFP@cargo micelles and their in vivo process on epilepsy models. (I) Ex vivo images of brains of normal and kindled mice with TFP@Cy5.5 administration. Latency to status epilepticus (J) and latency to generalized seizure (K) of epilepsy mice after different treatments. (H–K) Reproduced with permission from Zhang et al.^167^ Copyright 2023, Elsevier BV.

Epilepsy is a common neurological disorders characterized by recurrent seizures, affecting approximately 10 million people in China.^244^ The microenvironment of epilepsy can be described as excitotoxicity of glutamate and oxidative environment, as well as abnormal electrical activity of neurons. Based on the specific property of abnormal electrical activity, Ying et al.^161^ and Wang et al.^245^ developed electro‐responsive hydrogen nanoparticles (ANG–PHT–ERHNPs) for targeted delivery and on‐demand drug release of phenytoin sodium (PHT), a conventional antiepileptic drug, into the brain for epilepsy treatment (Figure 6A). The electro‐responsive nanoparticles (ERHNPs) were synthesized through a soap‐free emulsion copolymerization strategy (Figure 6B). Under an electric field, ERHNPs could swell owing to the ionization of sulfonate groups in their structure, resulting in increased particle size from 102.3 ± 16.8 to 388.0 ± 20.4 nm under the current of 500 μA within 1 min. In recent decades, PHT has been replaced as first‐line medication for epilepsy treatment due to its potential side effects at high dosage. Hence, specifical targeting of PHT to seizure focus is essential for improving antiepilepsy efficiency. After being loaded into ERHNPs, the release rate of PHT increased from 34.6 to 87.3% triggered by a current of only 200 μA (Figure 6C). These results suggested that these electro‐responsive ANG–ERHNPs have the capability for on‐demand drug release, while avoiding premature leakage.

Moreover, the biosafety of nanomaterials is of importance for drug delivery. Inspired by the electrical stimuli of epilepsy, Wu et al.^162^ reported a nanoengineered on‐demand drug delivery nanosystem capable of excellent electric‐responsiveness and biocompatibility for epilepsy therapy. This nanosystem was fabricated through one‐pot copolymerization of pyrrole and dopamine to form conductive polymer (PPY)–functionalized PDA nanomaterials (PPY–PDA). PHT and ANG were encapsulated and modified onto the PPY–PDA hybrid materials as antiepileptic drug and brain‐targeting peptide, respectively. The electrical conductivity of PPY was significantly enhanced with a dopamine mass ratio of 5%. In vitro drug release study showed the electric‐stimuli drug release behavior in response to the intermittent discharges. Interestingly, after three “on‐off” cycle of electric stimulus, PPY–PDA–PHT exhibited obvious drug release during the “on” state, while showing negligible drug leaking at “off” state, further implying their advantages of electro‐responsiveness in epilepsy treatment.

Micelles, consisting of a hydrophobic core and hydrophilic shell, have been widely utilized as nanocarriers for drug delivery, which can greatly improve the aqueous solubility of hydrophobic and insoluble drugs.^246^ In recent years, stimuli‐responsive micelles have gained considerable attention in the field of neurological disorders. For instance, Lu et al.^163^ constructed a ROS‐responsive polymeric micelle system (Ab–PEG–LysB/CUR) for AD treatment, by using an amphiphilic polymer (poly(ethylene glycol)(PEG)–LysB) as the ROS‐responsive section. After incubation with H_2_O_2_, these micelles were disassociated with irregular size distribution, which might be attributed to their conversion from amphiphilic to hydrophilic state in response to the oxidative environment associated with AD.^247^

#### 3.3.2 Imaging in neurological disorders

With the development of imaging technology, the clinical imaging of neurological disorders is largely hampered by the protection of BBB and the depth and accuracy of imaging.^248^ Hence, it is imperative to design nano‐imaging agents with high resolution and contrast, responsiveness to pathological environments, as well as BBB‐transporting ability for precise imaging of neurological disorders.

AD is one of the most occurring neurodegenerative disease characterized by progressive decline and cognitive capacity.^249^, ^250^ It has been reported that the concentration of metal ions (such as Cu^2+^, Fe^3+^, Al^3+^, etc.) in the AD environment is higher than that in normal tissue. Hence, detection of excessive metal ions holds potential for diagnosing and understanding the pathological conditions of AD.^251^ For instance, Wang et al.^164^ designed an activable PA probes (RPS1) for visualization of Cu^2+^ in AD brains (Figure 6D). As a small molecule, RPS1 with electron‐donating groups showed the longest absorption wavelength at 713 nm after specifically chelation with Cu^2+^ (Figure 6E). In vitro competition experiment showed that RPS1 displayed superior selectivity toward Cu^2+^ over other metal ions such as Fe^2+^, Fe^3+^, and Ni^2+^ and exhibited the strongest PA signal (Figure 6F). Inspired by these favorable results obtained from RPS1, in vivo PA imaging was demonstrated on AD mice (Figure 6G). Weak PA signals were observed in both normal mice brains following RPS1 injection, and AD mice treated with PBS. But a strong PA signal was captured specifically in the cortex region of AD mice after RPS1 treatment, proving that RPS1 could selectively detect Cu^2+^ concentration in the brain of AD mice through PA imaging.

Amyloid‐β (Aβ) plaques, assembled by Aβ monomers, are another pathological hallmark of AD, which will deposit to induce neurological disorders.^252^ Hence, the detection of Aβ plaques is another approach for predicting AD progression. Mao et al.^165^ reported an activatable NIR‐II fluorescent probes (DMP) for specifically binding to Aβ plaques. The fluorescence intensity of DMP was activated after incubation with Aβ fibrils, whereas no obvious change in fluorescence signal was observed with Aβ monomers or oligomers. Furthermore, both in vitro and in vivo NIR‐II fluorescence imaging showed the excellent Aβ‐activatable property of DMP. Notably, the fluorescence signal of DMP could be retained in the brain on AD mice reached up to 60 min, enabling real‐time longitudinal imaging in vivo.

#### 3.3.3 Theranostics in neurological disorders

MnO_2_ nanomaterials are one kind of the star biomaterials in the field of theranostics for neurological disorders, particularly in GBM treatment, due to their remarkable pH, ROS, and GSH‐responsive properties. The released Mn^2+^ can serve as MRI contrast agents for monitoring the drug delivery process, as well as enhancing therapeutic efficacy. Tan et al.^166^ designed a theranostic nanomaterial iRPPA@TMZ/MnO by loading drugs into MnO nanocarriers, and encapsulated them within polymeric micelles for glioma treatment. After systematic administration, iRPPA@TMZ/MnO can cross the BBB to reach the glioma site with the guidance from iRGD peptide targeting ligands. Upon excessive H_2_O_2_ levels at TME, iRPPA@TMZ/MnO will discompose to release TMZ, Mn^2+^, and O_2_, thereby improving therapeutic effects against glioma. Additionally, iRPPA@TMZ/MnO exhibited TME‐activatable T_1_‐weighted signal intensity under the trigger of acid pH and H_2_O_2_. After administration, the MRI signal in glioma site was stronger than normal site, indicating efficient tumor‐targeting and TME‐responsive properties of iRPPA@TMZ/MnO.

In our previous work, Zhang et al.^167^ fabricated an electro‐responsive nanomaterial by assembly Fc‐conjugated D‐a‐tocopherol polyethylene glycol succinate and amphiphilic Poloxamer 407, and encapsulating fluorescent dye Cy5.5 or antiepileptic drugs (TFP@cargo) for epileptic foci imaging and antiepileptic treatment (Figure 6H). The ex vivo fluorescence imaging showed that higher fluorescence intensity was observed on the epileptic foci compared with that on contralateral side in epilepsy mice (Figure 6I). Moreover, no obvious difference was obtained between both sides of the normal mice group. Besides, three antiepileptic drugs were utilized as substitutes for Cy5.5 in epilepsy treatment. The results showed that TFP micelles could improve the antiepileptic efficacy of drugs in two epilepsy models (Figure 6J,K). Combined with the results of electro‐responsive drug release, TFP@cargo could realize precise imaging of pathological sites, electro‐responsive drug release and enhanced antiepileptic effects.

The BBB, which serves as a protective shield against toxins and pathogen, poses a significant impediment to the delivery of nanomaterials for neurological disorders therapy. Therefore, improved BBB transport efficiency is imperative for enhancing the therapeutics of neurological disorders.

### 3.4 Inflammation

Inflammation is the defense mechanism in the body against various injurious factors. Prominent inflammatory diseases encompass periodontitis, inflammatory bowel diseases (IBDs), and rheumatoid arthritis (RA). Owing to the complex of inflammatory environment characterized by severe oxidative stress, acid condition, and overexpressed enzymes, the therapeutic efficacy of inflammatory diseases is largely compromised.^253^ Therefore, stimuli‐responsive nanomaterials are emerging with the requirement of precise treatment.

#### 3.4.1 Drug delivery in inflammation

IBD is a chronic and nonspecific inflammation that influence the mucosa and submucosa of the large intestine. Aminosalicylates, steroids, immunosuppressants and biological agents are commonly used therapeutics in IBD therapy.^168^ As a synthetic steroid, budesonide (BDS) is applied in a number of inflammatory diseases. In order to solve the problem of poor solubility and quick elimination of BDS, Sun et al.^168^ proposed a pH/redox dual‐responsive nanomaterial (ATP–CMI) for efficient BDS delivery. This nanomaterial was obtained by self‐assembly of carboxymethyl inulin (CMI) modified with 4‐aminothiophenol (4‐ATP) derivatives via aromatic moieties. In vitro drug release results showed that more than 80 wt% BDS was released in the presence of GSH at pH 6.0, which was higher than in the GSH‐free physiological conditions. The mechanism of pH/ROS‐responsive manner might be due to the cleavage of disulfide bonds within the nanomaterial structure. The treatment efficiency against colitis was investigated using a dextran sulfate sodium (DSS)‐induced colitis model. Following BDS‐NPs treatment, the mice displayed reduced colon weight‐to‐length ratio, less weight loss, and decreased histological scoring compared with PBS‐treated mice and BDS‐sus‐treated mice. These improvements might be attributed to targeted accumulation of BDS‐NPs at inflammatory regions, followed by the pH/ROS‐responsive drug release for enhanced drug delivery.

Periodontitis is a chronic inflammatory disorder, affecting approximately 90% of population worldwide. Hydrogels are polymeric networks of hydrophilic polymer chains with high swelling and distending properties. Yu et al.^169^ designed a pH‐responsive chitosan‐based hydrogel loaded with N‐phenacylthiazolium bromide (PTB). In vitro drug release results showed that more PTB could be released at pH 5.5–6.5 compared with pH 7.4, demonstrating the excellent pH‐responsive ability of this hydrogel formulation. Besides, inflammatory cell infiltration and the loss of collagen matrix were distinctively reduced both in the induction and recovery phases. Moreover, this chitosan‐based hydrogel, with simple synthesis and good stimuli‐responsive property, is a promising nanomaterial for targeted drug delivery and on‐demand drug release in the treatment of inflammatory disorders.

#### 3.4.2 Imaging in inflammation

As an endogenous gas molecule, hydrogen sulfide (H_2_S) is intricately associated with a variety of diseases, such as inflammation, cancer, gastrointestinal, and so on.^254^, ^255^, ^256^ H_2_S is a double‐edged sword with protection or damage function against pathological processes. Monitoring H_2_S levels in the microenvironment is essential for comprehending pathological conditions. Liu et al.^170^ reported an innovative NIR‐II luminescent strategy by introducing absorption competition‐induced effect for real‐time imaging of hepatic inflammation. The NIR‐II luminescence probe (1‐PEI‐DCNPs) was synthesized by conjugation of H_2_S‐responsive chromophore with NIR‐II luminescent lanthanide nanoparticles using polyethylenimine (PEI). In the presence of H_2_S, the absorption peak at 820 nm of chromophore decreased, whereas the NIR‐II luminescence of nanoparticles at 1060 nm gradually recovered within 10 min, suggesting the good H_2_S‐responsive property of 1‐PEI‐DCNPs probe. Compared with NIR‐I, NIR‐II irradiation possesses longer wavelength, and therefore exhibit higher tissue penetration.^257^ Lipopolysaccharide‐induced liver inflammation was utilized as an ideal model to investigate the effectiveness for in vivo H_2_S detection. The results demonstrated that 1‐PEI‐DCNPs could sensitively reflect the damage and recovery of liver by H_2_S imaging, providing a distinct approach for detecting liver injury.

In another study, a chitosan‐based pH‐responsive nanomaterial was prepared by Jing et al. for acute pancreatitis (AP) imaging.^171^ The bimetallic ions of Ce and Ga were doped into carbon dots through the hydrothermal method. After that, chitosan was applied as a responsive nanocarrier for loading Ce/Gd–carbon dots and resveratrol (RES) loading. Encouraged by the pH‐responsive properties of chitosan, the carbon dots/RES@CS NPs (CRCS) could achieve responsive cargo release at inflammatory sites. The released carbon dots and Ce/Gd were employed for fluorescence and MR imaging, respectively, demonstrating the potential of CRCS in dual‐modal inflammation imaging.

#### 3.4.3 Theranostics in inflammation

Osteoarthritis (OA) is considered as the most common chronic joint disease characterized by acid environment and overexpressed MMP‐13.^258^, ^259^, ^260^, ^261^ Based on the unique microenvironment of OA, Lan et al.^172^ developed a pH/MMP‐13 dual‐responsive nanoplatform for OA theranostics. This nanoplatform was constructed by conjugating a pH‐responsive polymer (PPL) with an MMP‐13‐responsive peptide (H_2_N–GPLGVRGC–SH). To achieve fluorescence imaging, Cy5.5 dye was labeled onto the nanoplatform, and coupled with black hole quencher‐3 to effectively quench its fluorescence signal. The obtained pH/MMP‐13 dual‐responsive nanoplatform (MRC–PPL@PSO) exhibited excellent stability under physiological environment. Upon exposure to the acidic condition of OA, the nanoplatform was disassembled to release encapsulated drugs for anti‐inflammatory therapy. Besides, the overexpressed MMP‐13 in OA microenvironment cleaved the MMP‐13‐responsive peptide on the nanoplatform, triggering Cy5.5 release and subsequent fluorescence activation. Collectively, MRC–PPL@PSO has excellent dual‐responsiveness and potent antiarthritic effects.

Compared with traditional single‐photon fluorescence imaging, two‐photon excited fluorescence technology demonstrates superior dimensional resolution and enhanced tissue penetration capabilities.^262^, ^263^ Another theranostic compound has been constructed by bridging a two‐photon AIE fluorophore and anti‐inflammatory Pred via a ROS‐responsive linker.^173^ After packaged into polymeric micelles, the obtained TPP@PMM was capable with two‐photon bioimaging and ROS‐responsive release properties. After administration, a more obvious lesion of arthritis could be obtained using two‐photon confocal images than that of single‐photon imaging, proving that TPP@PMM is adaptable for two‐photon bioimaging and antiarthritis treatment, as well as theranostics for AS.

The acid pH and elevated ROS levels within the inflammatory microenvironment are commonly utilized triggers for stimuli‐responsive nanomaterials construction. However, it is crucial to address bacterial infection that often accompany inflammation.^19^

### 3.5 Bacterial infection

Bacterial infection is regarded as a significant threat to humanity, encompassing the pathological changes in human caused by the invasion of bacteria.^264^, ^265^ Aggressive surgical debridement and antibiotic therapy are the primary choices in antibacterial effect in clinic.^266^ However, the antibacterial efficiency of antibiotics is limited by the abuse of antibiotics and the formation of biofilms (protective defense of bacteria).^267^ With the development of nanotechnology, nanomaterials also display great potentials in antibacterial therapy. The microenvironment surrounding bacteria can be categorized into physical stimuli (temperature, light, and salt) and bacterial metabolites stimuli (acid, enzyme, and redox).^230^ Therefore, stimuli‐responsive nanomaterials have shown remarkable antibacterial effects through solving the conflict between biocompatibility and bactericidal efficiency by controlled drug release and inhibition of biofilms formation.^266^, ^268^, ^269^, ^270^ Therefore, stimuli‐responsive nanomaterials are suitable for antibacterial applications, including drug delivery and theranostics.

#### 3.5.1 Drug delivery in bacterial infection

Stimuli‐responsive nanomaterials have been widely utilized in drug delivery in bacterial infection owing to their ability to achieve on‐demand drug release and targeted properties at the infection regions.^271^ Drugs can be encapsulated in nanocarriers to improve their local concentrations and minimize systematic side effects. Bacteria infected microenvironment is considered to be acidic with the pH values ranging from 5.0 to 5.5 caused by the anaerobic glycolysis during metabolism.^272^ Hence, the slight acidic conditions associated with bacterial infections can serve as a switch for drug delivery. The design of pH‐responsive nanomaterials can also be realized through pH‐sensitive chemical bonds or linkers, pH‐triggered charge conversion, and pH‐responsive carriers.^273^ Sun et al.^174^ constructed a pH‐responsive antibacterial surface by coating polycationic polymer brushes and forming pH‐sensitive enamine bonds via yne‐amine click reaction. Once bacteria colonize on the surface of this nanomaterial, the pH‐sensitive bond would be broken down in response to local acid conditions leading to the exposure of polycations that exert antibacterial effects. In contrast, bacteria thrived wherever on the model strain of *Staphylococcus aureu* (*S. aureus*) or *Escherichia coli* (*E. coli*) in pH 7.4 conditions.

Methicillin‐resistant *Staphylococcus aureus* (MRSA) biofilms‐associated infection poses a significant threat to human health. As a defensive barrier, MRSA biofilms may undermine the penetration of antibiotics and cause local inflammation. To address this problem, Zhang et al.^175^ designed a pH‐responsive nanomaterial loaded with ICG as the photosensitizer (ICG–ZnS NPs). Upon acidic environment, ZnS NPs would decompose and release Zn^2+^ and H_2_S for cell metabolism prevention and gas therapy, respectively. Combined with ICG‐mediated hyperthermia therapy, this nanomaterial exhibit enhanced antibacterial efficacy both in vitro and in vivo, suggesting that ICG–ZnS NPs have great potentials as pH‐responsive nanomaterials for MRSA biofilms and wound infection eradiation.

#### 3.5.2 Imaging in bacterial infection

To effectively combat bacterial infection, it is of importance to timely diagnose and monitor infectious process. Nanomaterials integrating rapid and noninvasive diagnosis strategies have been developed for antibacterial therapy.^274^ Compared with “always‐on” nanomaterials, “turn‐on” nanomaterials triggered by bacterial microenvironment or exogenous stimulus can activate their own signal output for precise diagnosis.^275^ A variety of imaging strategies, such as MRI, CT, and US, have been developed to accurately distinguish bacterial infections in clinic.^276^, ^277^

As a noninvasive imaging approach, MRI has been exploring for the diagnosis of bacterial infection, especially utilizing distance‐dependent magnetic resonance tuning (MRET)‐based MRI, which can be activated by pathological changes. Inspired by the activation properties of MRET, Li et al.^180^ constructed a pH‐responsive MRET MRI probe (MDVG‐1) through the self‐assembly of paramagnetic enhancer (Gd–DNA_3_–Gd) and superparamagnetic quencher (MDV). The T_1_‐weighted signal was quenched in physiological environment. Under the acidic condition infected by bacteria, MDMG‐1 would disassemble into monomers, resulting in an increase in T_1_‐weighted signal (*r*_1_) of Gd–DNA_3_–Gd from 5.13 at pH 7.4 to 35.2 mM^−1^ s^−1^ at pH 5.5. In the meantime, the T_2_‐weighted signal (*r*_2_) of MDMG‐1 decreased from 187.1 to 86.2 mM^−1^ s^−1^. In vivo MRI images showed that MDVG‐1 had good accuracy for *S. aureus*‐infected site imaging. These results revealed that MDMG‐1 owned switchable abilities from T_2_ to T_1_‐weighted imaging under the bacterial infection, providing a potential approach for accurately diagnosing bacterial infection.

In another study, Li et al. proposed a peptide‐modified MRET probe (MPD‐1) via assembly of MNPs with Gd^3+^ modified MMP‐2 responsive peptide and bacteria‐targeting peptide.^181^ After exposure to MMP‐2 at the bacterial infection site, MPD‐1 disassembled into MNPs and Gd^3+^, and the *r*_2_/*r*_1_ ratio was decreased from 46.14 to 2.33, suggesting the switchable properties of MPD‐1 from T_2_ to T_1_‐weighted imaging owing to its response toward MMP‐2. Furthermore, after administration of MPD‐1 into *S. aureus*‐infected mice, the T_1_‐weighted signal in infection sites was significantly increased, whereas no obvious change in the sterile inflammation, demonstrating the excellent MMP‐2 responsive properties of MPD‐1.

#### 3.5.3 Theranostics in bacterial infection

Theranostics integrates real‐time diagnosis and in situ therapy into a single system for rapid, effective, and noninvasive detection and treatment.^278^ Bacterial biofilms, which are more resistant to antibiotics, can form during the development of bacterial infection.^279^, ^280^, ^281^ Hence, detection and eradication of bacteria in time could avoid the formation of biofilms, reduce the drug‐resistant of bacteria, and enhance antibacterial efficiency.^230^, ^282^ Liu et al.^182^ constructed a pH‐responsive theranostic nanomaterial through self‐assembly of poly(poly(ethylene glycol) methyl ether methacrylate)‐*b*‐poly(2‐(diisopropylamino) ethyl methacrylate‐*co*‐2‐hydroxyethyl methacrylate)–chlorin e6 (PPEGMA‐*b*‐P(DPA‐*co*‐HEMA)–Ce6). Under pH 6.0 condition, PPEGMA‐*b*‐P(DPA‐*co*‐HEMA)–Ce6 NPs underwent a charge conversion from negative of −1.45 mV at pH 7.4 to highly positive of +11.6 mV, which could be ascribed to the protonation of PDPA ligand. The nanoparticle size also increased from 78 to 90 nm with the decrease of pH value. In addition, the pH‐dependent interaction of nanoparticles with *E. coli* bacteria has been investigated via flow cytometry. The results showed that higher fluorescence signals could be observed after NPs treatment under pH 6.2, whereas no obvious fluorescence change was obtained after free Ce6 incubation at pH 6.0–7.4. These results demonstrated that PPEGMA‐*b*‐P(DPA‐*co*‐HEMA)–Ce6 NPs exhibited excellent pH‐responsive properties for on‐demand binding with *E. coli* and fluorescence imaging. Besides, owing to the synergetic effects of the cationic property of P(DPA‐*co*‐HEMA)–Ce6 and Ce6‐mediated PDT, PPEGMA‐*b*‐P(DPA‐*co*‐HEMA)–Ce6 NPs demonstrated effective antibacterial activity in an acidic environment.

Aggregation‐induced emission (AIE)‐based nanomaterials are capable of overcoming aggregation‐caused quenching effect of traditional fluorescence probes, thereby enabling ROS generation for bacterial imaging and ablation.^283^ In order to specifically track the in vivo process of bacteria and precisely kill bacteria, H_2_O_2_‐responsive MIL‐100 (Fe) NPs were utilized for delivering 3‐azido‐_D_‐alanine (_D_‐AzAla).^183^ In the presence of H_2_O_2_ (50 μM) simulated inflammatory environment of bacteria, about 60% of _D_‐AzAla was released within 10 h, which was higher than that incubated with low H_2_O_2_ levels of 0.5 μM in physiological condition. The TEM images and increased size of NPs also illustrated the H_2_O_2_‐responsive properties of NPs. After that, AIE photosensitizer PS NPs were injected to react with bacteria through in vivo click chemistry. The released _D_‐AzAla selectively integrated into the bacteria and exhibited fluorescence upon reaction with DBCO‐Cy5 for fluorescence imaging. With the assistance of PDT, the number of bacteria was sharply reduced. This study provided a bacteria metabolic labeling strategy for timely diagnosis and precise antibacterial therapy.

The applications of stimuli‐responsive nanomaterials displayed great promise in antibacterial therapy, including ameliorating the resistance and sides effects of antibiotics. However, these nanomaterials are still in their early stages, necessitating the resolution of several challenges including technology limitations, appropriate antibiotic selection, and nanomaterial toxicity.^269^

## 4 CONCLUSION AND OUTLOOK

In recent years, stimuli‐responsive nanomaterials have gained increasing attention within the field of biomedicine and nanomedicine. Either endogenous or exogenous stimuli‐responsive modality possesses unique peculiarity for drug delivery and release. Compared with nonresponsive nanomaterials, stimuli‐responsive nanomaterials, also known as “smart” nanomaterials, offer effective and promising approaches to improved bioavailability. These nanomaterials enable on‐demand drug release at tumor site with minimal dosages of therapeutics, thereby reducing side effects in the meantime.

In this review, we summarize the design, sensitive mechanism, pros and cons of stimuli‐responsive nanomaterials in response to both endogenous and exogenous stimulus. These nanomaterials display the potentials in on‐demand drug release at specific sites, enhanced therapeutic efficacy and reduced side effects of free drugs. Based on that, the latest biomedical applications of these nanomaterials, including CVDs, cancer, neurological disorders, inflammation, and bacterial infection, are presented, showcasing that stimuli‐responsive nanomaterials hold promise as effective platforms for drug delivery, imaging, and theranostics.

Currently, stimuli‐responsive nanomaterials have shown promising effects in preclinical animal models, whereas only a few of them have been applied in clinical trials (Table 3).^9^ It reminds us that there are still considerable challenges of stimuli‐responsive nanomaterials should be taken into consideration.

**TABLE 3:** Stimuli‐responsive nanomaterials for biomedical applications in clinical trials.

Stimuli	Diseases	Nanomaterials	Identifier	Clinical status
pH	Lung cancer	Chitosan	NCT04218188	Observational
Temperature	Hepatocellular carcinoma	ThermoDox	NCT00617981	Phase III
Light, ultrasound and magnetic field	AS	Silica–gold iron‐bearing nanoparticles	NCT01270139	Not applicable
Magnetic field	Liver cancer	Magnetic beads–DOX (MTC–DOX)	NCT00034333	Phase II Phase III
Ultrasound	Intrabony defects	Nanohydroxyapatite	NCT03399279	Not applicable

*Note*: Data were obtained from ClinicalTrials.gov.

First, toxicity and biosafety of stimuli‐responsive nanomaterials pose major concerns for their biomedical application. Compared with local administration, systematical administration is more noninvasive and will not induce local inflammation. When administered systematically, circulation time, toxicity, and adverse off‐target effects of nanomaterials should also be considered. Additionally, most studies on stimuli‐responsive nanomaterials remained at the animal experiment step. Thus, further systematic investigations should be carried out for preclinical evaluation, and the in vivo stability and degradation process should also be predetermined. Therefore, a comprehensive understanding of the circulation dynamics, release kinetics, and degradation behavior of nanomaterials in the body after systematical administration is important for precise control o transport and clearance of nanomaterials, minimizing their potential toxicity toward normal tissues.^284^

The cost and complexity associated with the synthesis process of stimuli‐responsive nanomaterials is also a hurdle on the way to translation. Currently, the responsive property and the therapeutic efficacy are limited to laboratory experiments, making it challenging to expand the production on a large scale using a simplified method, as well as lower the cost. Hence, there is still a long way to go from laboratory experiments to clinical trials.

Last but not least, the controlled drug release behavior also plays an important role in the fabrication of nanomaterials. The flexibility of pathological environment provides more potentials for researchers to design stimuli‐responsive nanomaterials for drug delivery, diseases diagnosis, and treatment. However, the responsive release manner of drugs is hampered by the response sensitivity of nanomaterials, as well as the content of endogenous substances within the pathological environment. Therefore, specific environment to which nanomaterials respond and the stimuli sensitivity of nanomaterials play crucial roles in determining their drug release behavior, biodistribution, and therapeutic efficacy.

In all, further attention should focus on developing stimuli‐responsive nanomaterials with low toxicity, facile preparation, on‐demand drug release, and enhanced therapeutic efficacy, thereby providing novel strategies for biomedical applications.

## AUTHOR CONTRIBUTIONS

*Literature review; writing—original draft preparation and figures*: Xiaojie Chen. *Review and editing*: Di Wu and Zhong Chen. *Conceptualization*: Di Wu and Zhong Chen. *Supervision*: Zhong Chen. All authors read and approved the final manuscript.

## CONFLICT OF INTEREST STATEMENT

There is no conflict of interest in this study.

## ETHICS STATEMENT

Not applicable.

## Data Availability

Not applicable.
